# A systematic review of the health-related quality of life and economic burdens of anorexia nervosa, bulimia nervosa, and binge eating disorder

**DOI:** 10.1007/s40519-016-0264-x

**Published:** 2016-03-04

**Authors:** Tamás Ágh, Gábor Kovács, Dylan Supina, Manjiri Pawaskar, Barry K. Herman, Zoltán Vokó, David V. Sheehan

**Affiliations:** 1 Syreon Research Institute, 119 Thököly Street, 1146 Budapest, Hungary; 2 Formerly of Shire, 300 Shire Way, Lexington, MA 02421 USA; 3 Shire, 300 Shire Way, Lexington, MA 02421 USA; 4 Department of Health Policy and Health Economics, Faculty of Social Sciences, Eötvös Loránd University, 1/a Pázmány Péter Street, 1117 Budapest, Hungary; 5 University of South Florida College of Medicine, 12901 Bruce B. Downs Boulevard, Tampa, FL 33612 USA

**Keywords:** Eating disorders, Quality of life, Cost of illness, Anorexia nervosa, Bulimia nervosa, Binge eating disorder

## Abstract

**Purpose:**

To perform a systematic review of the health-related quality of life (HRQoL) and economic burdens of anorexia nervosa (AN), bulimia nervosa (BN), and binge eating disorder (BED).

**Methods:**

A systematic literature search of English-language studies was performed in Medline, Embase, PsycINFO, PsycARTICLES, Academic Search Complete, CINAHL Plus, Business Source Premier, and Cochrane Library. Cost data were converted to 2014 Euro.

**Results:**

Sixty-nine studies were included. Data on HRQoL were reported in 41 studies (18 for AN, 17 for BN, and 18 for BED), on healthcare utilization in 20 studies (14 for AN, 12 for BN, and 8 for BED), and on healthcare costs in 17 studies (9 for AN, 11 for BN, and only 2 for BED). Patients’ HRQoL was significantly worse with AN, BN, and BED compared with healthy populations. AN, BN, and BED were associated with a high rate of hospitalization, outpatient care, and emergency department visits. However, patients rarely received specific treatment for their eating disorder. The annual healthcare costs for AN, BN, and BED were €2993 to €55,270, €888 to €18,823, and €1762 to €2902, respectively.

**Conclusions:**

AN, BN, and BED have a serious impact on patient’s HRQoL and are also associated with increased healthcare utilization and healthcare costs. The burden of BED should be examined separately from that of BN. The limited evidence suggests that further research is warranted to better understand the differences in long-term HRQoL and economic burdens of AN, BN, and BED.

**Electronic supplementary material:**

The online version of this article (doi:10.1007/s40519-016-0264-x) contains supplementary material, which is available to authorized users.

## Introduction

Anorexia nervosa (AN), bulimia nervosa (BN), and binge eating disorder (BED) are prevalent psychiatric disorders that are characterized by different symptoms. Based on mixed gender surveys, the lifetime prevalence of AN is estimated to be approximately 0.5–0.6 %, that of BN 0.5–1 %, and that of BED 1.1–2.3 % [1–3]. The onset of the majority of eating disorders (EDs) occurs between the ages of 10–20 years; however, in contrast to AN and BN, BED often occurs in older cohorts [3]. AN, BN, and BED are associated with numerous physical (e.g., diabetes, hypertension, ulcers) and mental health conditions (e.g., anxiety disorder, depression) [2, 4–7]. Furthermore, patients with EDs have significantly elevated mortality rates compared with the standard population norms as well as higher suicide rates [8, 9].

Despite the major public health burden of AN, BN, and BED, there is lack of evidence on the differences in the health-related quality of life (HRQoL) and economic impact of different EDs [10, 11]. The objective of this study is to perform a systematic review of the published literature on the HRQoL and economic burdens of AN, BN, and BED. To our knowledge, no systematic review has been published on this topic to date.

## Materials and methods

This systematic review was based on an extensive literature search on the epidemiology, and HRQoL and economic burdens of EDs (i.e., AN, BN, BED, and eating disorders not otherwise specified [EDNOS]). The literature search was conducted in 2013 using Medline, Embase (via Scopus), PsycINFO, PsycARTICLES, Academic Search Complete, CINAHL Plus, Business Source Premier (via Ebsco Host), and Cochrane Library. The search terms are detailed in Online Resource 1. The search results were processed in two steps: first, the titles and abstracts of all the articles were screened; next, all the potentially relevant articles were analyzed in full text. The references of the included articles were screened for additional eligible studies. The literature screening was conducted by two independent reviewers and disagreements were resolved by the principal researcher.

The reasons for exclusion were as follows for the articles: (1) was not written in English; (2) was not published in a peer-reviewed journal; (3) was an editorial, letter, case report, or review; (4) was not specific to AN, BN, or BED; and (5) had an objective other than studying the HRQoL and/or economic burden (i.e., healthcare resource utilization, healthcare costs, and societal costs) of AN, BN, or BED. This review includes only publications in which AN, BN, and BED samples were clearly separated.

The following information was extracted from each included study: (1) the first author and year of publication; (2) the country; (3) the study design; (4) the study year; (5) the included ED(s); (6) the diagnostic criteria for EDs; (7) the characteristics of the study sample [sample size, % of female, age, and body mass index (BMI)]; (8) data on the HRQoL burden (HRQoL instrument, main findings); (9) data on healthcare utilization (i.e., resource use data on primary care, outpatient services, and inpatient care including emergency services); and (10) data on healthcare costs (i.e., the costs generated by health service utilization and medication use) and/or societal costs (i.e., costs related to the loss of productivity and symptom-related food costs) (perspective of the analysis, year of pricing, resource use and/or cost categories, and resource use and/or cost data).

To compare costs across studies, the costs were extrapolated to annual costs per case and inflated to year 2014 values, using country-specific GDP (gross domestic product) deflators. The costs were converted into United States (US) dollars where necessary, using purchasing power parities and then exchanged to euro (€) using the average annual US dollar/euro exchange rate for 2014 (0.7536). If the year of pricing was not referenced, the midpoint in the observation period was assumed to be the base year; if no observation period was reported, the year of publication was adopted as the base year.

## Results

**Fig. 1:**
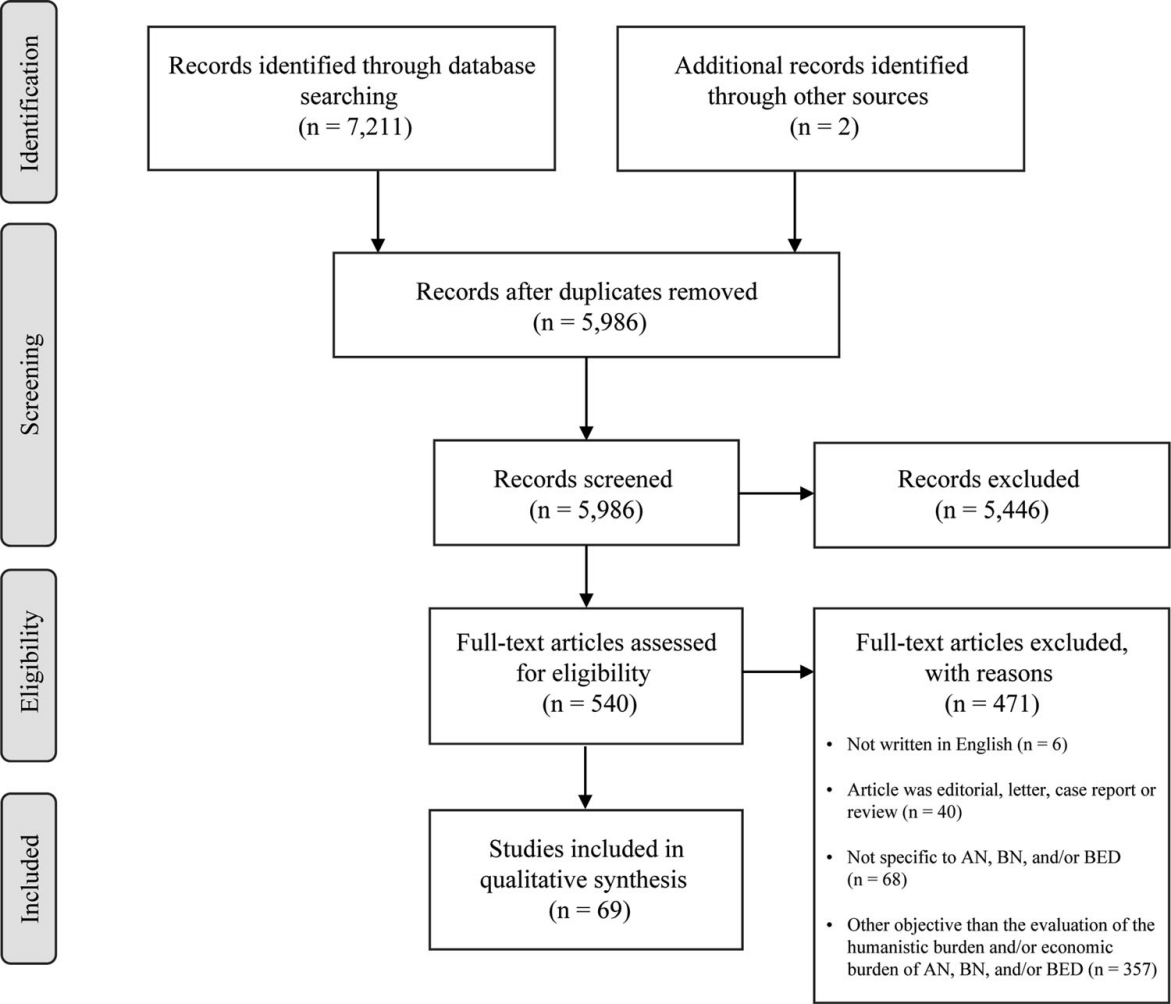
The flow diagram of the systematic literature search

The systematic literature search resulted in 7211 hits. The screening of titles and abstracts identified 540 potentially eligible articles. Finally, 69 studies were included in this review. Two records were identified through a hand search of the references of the relevant articles. A flow diagram of the systematic literature search, based on the Preferred Reporting Items for Systematic Reviews and Meta-Analyses (PRISMA) template [12], is presented in Fig. 1. The characteristics of the reviewed studies are detailed in Online Resource 2.

### Health-related quality of life burden

Data on the HRQoL of AN, BN, BED, and relatives/caregivers of ED patients were reported in 41 studies: 18 for AN [13–30], 17 for BN [13–15, 17, 20–25, 27, 29–34], 18 for BED [17, 21, 22, 24, 35–48], and 5 for the relatives/caregivers of ED patients [49–53]. Seventeen types of HRQoL instruments were applied in the included studies. The Medical Outcome Study Short Forms (i.e., Short Form 36 [SF-36] and Short Form 12 [SF-12]) were the most commonly used (*n* = 19) HRQoL questionnaires and were administered alone [16, 17, 19, 21, 24–27, 32, 33, 36, 38, 40, 42, 43] or in combination with other HRQoL questionnaires [13, 22, 23, 37] (Table 1). The reported HRQoL data are summarized in Online Resource 3.

**Table 1:** SF-12 and SF-36 scale and summary scores of AN, BN, and BED samples

Instrument	First author	Study group	Physical functioning	Physical role	Bodily pain	General health	Vitality	Social functioning	Emotional role	Mental health	PCS	MCS
			Mean (SD)	Mean (SD)	Mean (SD)	Mean (SD)	Mean (SD)	Mean (SD)	Mean (SD)	Mean (SD)	Mean (SD)	Mean (SD)
AN												
SF-36	del Valle [16]	Training^a^	93.2 (7.5)	67.6 (21.1)	95.6 (7.4)	70.7 (14.5)	76.7 (15.3)	66.1 (18.7)	72.5 (24.3)	73.2 (14.5)	58.8 (14.0)	44.2 (9.4)
		Control^a^	83.2 (17.6)	45.6 (39.8)	59.7 (26.5)	52.2 (21.0)	51.3 (33.5)	49.4 (28.3)	56.8 (32.0)	40.2 (25.2)	44.3 (11.5)	35.1 (16.8)
	Doll [17]	AN	88.92 (11.40)	73.75 (34.42)	63.30 (21.48)	53.26 (19.01)	41.63 (19.15)	72.37 (20.38)	49.38 (42.03)	52.57 (17.27)	43.40 (9.02)	45.54 (8.91)
	Gonzalez-Pinto [19]	AN	83.98 (13.2)	61.70 (40.3)	72.91 (25.6)	57.42 (20.4)	52.34 (23.6)	63.56 (29.9)	65.95 (40.2)	49.29 (23.5)	50.41 (8.2)	36.25 (14.6)
	Padierna [24]	AN-P	88.9 (16.4)	52.1 (43.3)	64.9 (30.1)	50.1 (23.1)	44.5 (23.5)	51.5 (30.1)	41.7 (42.8)	41.5 (23.4)	n.r.	n.r.
		AN-R	87.5 (15.4)	62.0 (40.6)	72.4 (22.7)	51.6 (19.1)	51.7 (22.0)	62.3 (30.9)	57.6 (42.3)	47.8 (23.2)	n.r.	n.r.
	Rie [25]	AN	80.2 (18.2)	42.0 (37.7)	65.9 (23.1)	48.8 (21.3)	39.5 (17.7)	46.6 (22.5)	29.5 (36.8)	41.6 (16.6)	n.r.	n.r.
	Turner [27]	AN	68.9 (21.5)	14.3 (25.4)	42.0 (22.0)	35 (18.8)	18.2 (14.9)	32.5 (24.8)	21.4 (28.0)	30.9 (22.0)	n.r.	n.r.
SF-12	Mond [22]	AN-P	n.r.	n.r.	n.r.	n.r.	n.r.	n.r.	n.r.	n.r.	46.80 (10.03)	26.96 (7.44)
		AN-R	n.r.	n.r.	n.r.	n.r.	n.r.	n.r.	n.r.	n.r.	45.38 (10.28)	38.38 (11.05)
BN												
SF-36	Doll [17]	BN	90.62 (11.90)	67.71 (35.99)	72.44 (22.46)	53.98 (19.93)	42.85 (20.05)	70.51 (21.32)	35.47 (43.95)	51.84 (18.05)	47.20 (9.47)	43.76 (9.35)
	Nickel [33]	Topiramate^a^	67.2 (6.5)	57.1 (6.0)	59.8 (7.2)	51.0 (5.3)	52.3 (4.1)	64.9 (6.2)	61.2 (8.0)	60.9 (6.1)	n.r.	n.r.
		Placebo^a^	65.4 (5.8)	57.1 (5.3)	61.5 (7.0)	53.6 (5.5)	53.3 (5.3)	64.1 (7.7)	57.6 (8.2)	60.4 (6.2)	n.r.	n.r.
	Padierna [24]	BN	89.5 (11.9)	54.8 (41.4)	63.3 (28.0)	47.1 (21.0)	44.9 (20.8)	54.5 (28.1)	41.8 (43.6)	46.4 (23)	n.r.	n.r.
	Rie [25]	BN	84.5 (15.4)	44.6 (39.2)	67.8 (19.6)	52.5 (17.5)	36.2 (15.7)	42.2 (26.4)	22.2 (32.6)	38.3 (16.9)	n.r.	n.r.
	Turner [27]	BN	80.0 (25.4)	57.1 (44.0)	60.8 (24.7)	42.9 (22.0)	27.4 (17.1)	49.1 (30.6)	30.8 (37.9)	34.2 (17.8)	n.r.	n.r.
SF-12	Mond [22]	BN	n.r.	n.r.	n.r.	n.r.	n.r.	n.r.	n.r.	n.r.	49.25 (10.07)	27.60 (9.44)
	Mond [32]	OBEs	n.r.	n.r.	n.r.	n.r.	n.r.	n.r.	n.r.	n.r.	46.50 (10.21)	36.34 (11.98)
		SBEs	n.r.	n.r.	n.r.	n.r.	n.r.	n.r.	n.r.	n.r.	48.84 (10.82)	36.28 (12.23)
		OBEs and SBEs	n.r.	n.r.	n.r.	n.r.	n.r.	n.r.	n.r.	n.r.	45.77 (11.64)	36.58 (10.86)
BED												
SF-36	de Zwaan [36]	Postoperative	82.8 (16.4)	61.1 (28.3)	55.3 (26.0)	42.4 (30.9)	41.7 (25.9)	72.4 (31.5)	92.6 (22.3)	59.1 (24.9)	42.2 (8.9)	45.7 (10.4)
	de Zwaan [37]	BED	36.7 (13.3)	23.5 (21.2)	39.1 (13.9)	39.1 (16.7)	19.4 (13.3)	51.4 (14.9)	53.6 (43.2)	68.4 (13.1)	28.1 (6.8)	44.7 (9.5)
	Doll [17]	BED	87.23 (11.55)	52.49 (34.92)	72.63 (21.79)	55.83 (19.27)	42.12 (19.43)	72.28 (20.68)	23.78 (42.57)	52.78 (17.51)	46.31 (9.12)	40.83 (9.00)
	Faulconbridge [38]	Bariatric surgery^a^	34.9 (1.9)	41.8 (1.7)^b^	39.5 (1.6)^b^	38.6 (1.7)^b^	39.0 (1.6)^b^	38.3 (2.0)^b^	42.3 (1.7)^b^	43.5 (1.8)^b^	37.7 (1.7)^b^	43.1 (1.6)^b^
		Lifestyle modification^a^	37.3 (1.5)	43.7 (1.5)^b^	44.8 (1.6)^b^	41.4 (1.4)^b^	42.7 (1.4)^b^	42.7 (1.7)^b^	43.7 (1.8)^b^	44.8 (1.7)^b^	40.8 (1.3)^b^	45.4 (2.0)^b^
	Marchesini [42]	CBT	68 (23)	60 (40)	60 (28)	55 (12)	51 (21)	63 (25)	59 (41)	58 (21)	43 (10)	42 (13)
		Control	71 (23)	64 (40)	61 (27)	57 (10)	51 (20)	64 (26)	57 (41)	59 (21)	44 (10)	42 (12)
	Masheb [43]	BMI ≥30 kg/m^2^	69.9 (21.8)	63.4 (39.8)	56.7 (23.7)	62.4 (18.8)	37.6 (19.5)	63.3 (27.6)	49.8 (42.1)	58.2 (15.9)	45.3 (9.6)	39.3 (10.6)
		BMI <30 kg/m^2^	91.5 (8.5)	79.3 (35.1)	72.8 (22.8)	74.7 (14.6)	47.2 (19.9)	77.2 (19.8)	60.9 (42.2)	62.3 (18.6)	53.6 (9.4)	41.0 (12.2)
	Padierna [24]	BED	72.3 (22.7)	46.9 (42.7)	60.7 (30.9)	50.7 (23.1)	40.9 (21.5)	52.2 (25.9)	37.3 (45.5)	44.5 (16.0)	n.r.	n.r.
SF-12	Mond [22]	BED	n.r.	n.r.	n.r.	n.r.	n.r.	n.r.	n.r.	n.r.	40.18 (13.11)	30.36 (7.96)

Only studies in which eating disorder-specific SF-12/SF-36 scale and/or summary scores were presented are included in Table 1

*AN* anorexia nervosa, *AN-P* anorexia nervosa, purging type, *AN-R* anorexia nervosa, restricting type, *BED* binge eating disorder, *BMI* body mass index, *BN* bulimia nervosa, *CBT* cognitive behavioral therapy, *MCS* mental component summary, *n.r.* not reported, *OBEs* objective bulimic episodes, *PCS* physical component summary, *SBEs* subjective bulimic episodes, *SD* standard deviation, *SF-12* Medical Outcomes Study Short-Form 12, *SF-36* Medical Outcomes Study Short-Form 36

^a^Baseline data; ^b^mean (standard error)

Patients with AN, BN, and BED were shown to have significantly lower HRQoL than the general population [18, 34, 37, 39, 40, 43]. Many of the included studies investigated the differences between the HRQoL impact of AN, BN, and BED/EDNOS. One of these studies evaluated patients with AN or BN and compared them to healthy subjects [20]. Both ED groups showed significantly more impairment than controls in the health domains of the Nottingham Health Profile (NHP), and the AN patients had significantly reduced mobility compared with the BN patients and the healthy controls. Three studies compared patients with AN, BN, or BED. One of them showed no differences in HRQoL, but demonstrated a significant relationship between HRQoL and the severity of eating symptomatology [24]. Another study found a more heterogeneous picture using the SF-36 [17]. Patients with BN or BED had lower scores than the non-ED subjects on the SF-36 Mental Component Summary (MCS) (mean [SD]: 43.76 [9.35], 40.83 [9.00] and 49.83 [16.47], respectively), but no differences were found on the SF-36 Physical Component Summary (PCS) between the ED groups and the non-ED group (mean [SD]: 43.40 [9.02] for AN, 47.20 [9.47] for BN, 46.31 [9.12] for BED, and 48.08 [16.67] for non-ED subjects). Regarding the various domains, the BN patients had lower scores on the Role Emotional, Social Functioning, Mental Health, Vitality, and General Health scales than the non-ED subjects, and the BED patients had lower scores on the Role Emotional, Mental Health, and Vitality scales than the non-ED subjects [17]. Mond et al. [22] presented similarly heterogeneous findings: significantly lower MCS scores in the BN and purging AN groups compared to the restrictive AN group (the MCS mean [SD] scores were: 27.60 [9.44] vs. 26.96 [7.44] vs. 38.38 [11.05], respectively), and significantly lower World Health Organization Brief Quality of Life Assessment (WHOQOL-BREF) Social Relationship Scale (QoLS) scores in the BED and purging AN groups compared with the restrictive AN group (mean [SD]: 2.20 [0.98] vs. 2.58 [1.06] vs. 3.58 [1.01], respectively). Rie et al. [25] could not show any HRQoL difference on comparing AN, BN, and EDNOS groups to each other, but all of the ED patients had significantly poorer HRQoL than the normal reference group. Focusing on EDNOS [27], no differences were detected between the EDNOS and BN groups on the SF-36 General Health and Healthy Status scales, but AN was associated with lower scores on the Social Functioning, Vitality, and Physical Functioning scales than EDNOS. Bamford et al. [15] found lower Psychological and Physical/Cognitive Scale scores (using the Eating Disorder Quality of Life [EDQoL] instrument) in AN than in BN and EDNOS. Latner et al. [21] found that SF-36 scores were worse in those with subjective bulimic episodes, food avoidance, laxative abuse, and self-induced vomiting, and that PCS scores were worse in those with subjective bulimic episodes and food avoidance. However, these authors did not find differences in HRQoL between AN, BN, BED, and EDNOS.

Eight studies investigated the separate and joint effects of BED and obesity (defined as BMI ≥30 kg/m^2^) on HRQoL [36, 37, 40, 41, 43–46]. The results of Masheb et al. [43] showed that BED patients with obesity had significantly lower scores on several SF-36 subscales (i.e., Physical Functioning, Bodily Pain, General Health, Vitality, Social Functioning, and PCS) than those without obesity (the PCS mean [SD] scores in patients with BMI ≥30 kg/m^2^ and BMI <30 kg/m^2^ were: 45.3 [9.6] vs. 53.6 [9.4], respectively, *P* = 0.001). Among obese individuals, all but two studies [41, 45] concluded that BED patients had significantly reduced HRQoL compared with individuals without BED [36, 37, 46], even in a population with extreme obesity (defined as BMI ≥40 kg/m^2^) [40] (Online Resource 3). Perez et al. [44] found that obesity was more strongly related to physical HRQoL variables, but BED was more predictive of the mental health and social functioning HRQoL variables. In contrast, Ricca et al. [45] did not find any significant differences in HRQoL between those obese patients without BED, those with threshold, and those with subthreshold BED (defined by a minimum average binge eating [BE] frequency of once a month for a minimum duration of 6 consecutive months). In the study conducted by Kolotkin et al. [41], BED was shown to be associated with more impaired HRQoL in obese individuals (the IWQOL mean (SD) total scores in the BED and non-BED patients were: 51.5 (21.9) vs. 65.3 (19.8), respectively, *P* < 0.001), but BED did not prove to be an independent factor of weight-related quality of life after controlling for BMI, demographic variables, and psychological variables.

The HRQoL burden of EDs is determined not only by the deficit in HRQoL of the patients themselves, but also by the potential deficit of close relatives who live with the patient or caregivers and take care of an ED patient. A study found that 80 % of the siblings of adolescent ED patients reported that their HRQoL was negatively affected by the onset of their siblings’ ED [49]. De La Rie et al. [50] showed that the caregivers of ED patients had worse HRQoL than normal controls (mean SF-36 scale scores: 56.2 [Vitality] to 90.4 [Physical Functioning] vs. 68.6 [Vitality] to 84.0 [Social Functioning], respectively). The caregivers’ perceived burden improved significantly over the first year of follow-up, but no further improvement was observed with longer follow-up [51]. Many factors were associated with higher caregiver burden, such as higher anxiety and depression, purgative behaviors, lower patient HRQoL [52], and low-level education [53].

### Economic burden

Twenty studies reported data on healthcare utilization: 14 for AN [3, 54–66], 12 for BN [2, 3, 34, 54, 58–62, 64–67], and 8 for BED [2, 3, 39, 59, 65, 66, 68, 69]. The healthcare utilization data extracted from the included studies are provided in Online Resource 4.

AN (78 %), BN (88 %), and BED (73 %) were associated with increased health service use (for any treatment, lifetime) compared with individuals without an eating disorder (44 %) [66]. In the study conducted by Striegel-Moore et al. [69], the number of 12-month total health service days (i.e., inpatient care, outpatient care, and emergency care) of BED patients (11.8–21.4) was higher than in the healthy comparison group (3.4–8.4), but it was similar to the utilization found in other major psychiatric disorders (6.9–18.4). The health service use in AN was reported to be equal to or higher than in BN or BED [3, 54, 58–62, 64, 65]. The difference in healthcare utilization between the AN and BN patients was largest in the case of hospitalization. The length of hospital stay was found to be much longer for patients with AN (15.0–52.7 days) than for those with BN (9.0–45.7 days) [54, 58, 60, 61, 64].

Only one study evaluated the effect of obesity (defined as BMI ≥30 kg/m^2^) on health service use in patients with BED. In the study by Striegel-Moore et al. [69], the number of 12-month total health service days (i.e., inpatient care, outpatient care, and emergency care) was found to be higher for obese BED women (mean [SD] total service days: 21.4 [28.1]) than for nonobese BED women (mean [SD] total service days: 11.8 [21.8]); however, the association between obesity and health service use was not significant.

In the assessed studies, patients with AN, BN, or BED rarely received treatment for their ED, but received more frequent treatment for comorbid psychiatric symptoms and/or weight loss [2, 3, 59, 66]. In the study conducted by Mond et al. [59] on AN, BN, and BED, 22, 14, and 23 % of the patients, respectively, were treated by a mental health professional, specifically for their ED in their lifetime.

Data on healthcare costs were reported in 17 studies: 9 for AN [58, 60, 61, 70–75], 11 for BN [31, 34, 58, 60, 61, 73, 75–79], and 2 for BED [39, 68]. In the reviewed studies, the annual healthcare costs for AN, BN, and BED ranged from €2993 [61] to €55,270 [71], €888 [78] to €18,823 [79], and €1762 [39] to €2902 [68], respectively (Table 2). Detailed information on the reported healthcare costs is presented in Online Resource 5.

**Table 2:** Annual cost per patient data on AN, BN, and BED

First author	Perspective	Country	Annual cost per patient in 2014 EUR	
AN				
Byford [70]	Payer	UK	Inpatient care	€23,607
			Specialist outpatient care	€18,280
			General outpatient care	€27,889
			Included cost categories: inpatient nights, outpatient appointments, day patient contacts, accident and emergency contacts, state day school, independent day school, independent boarding school, hospital school, home tuition, school counselor, education welfare officer, general practitioner, practice nurse, dietician, district nurse, health visitor, community pediatrician, community psychiatric nurse, clinical psychologist, counselor, family therapist, dentist, school doctor, school nurse, social worker, eating disorders association, family therapy, foster care	
Crow [71]	Payer	USA	Usual care	€16,785
			Adequate care	€55,270
			Included cost categories: inpatient treatment, partial hospitalization, psychotherapy, medication management, fluoxetine	
Haas [72]	Hospital	Germany	€4,900 Included cost categories: physicians, nursing, psychotherapists, dietician, art and music therapy, psychotherapy, drugs, medical products, services on demand, overhead costs (medical/non-medical infrastructure)	
Haas [73]	Hospital	Germany	€5,445 Included cost categories: physicians, nursing, psychotherapists, dietician, art and music therapy, psychotherapy, drugs, medical products, services on demand, overhead costs (medical/non-medical infrastructure)	
Krauth [58]	Societal	Germany	€5,952 Included cost categories: inpatient treatment, convalescence statutory health insurance, rehabilitation statutory pension insurance, inability to work, death	
Lock [74]	Payer	USA	€30,700 Included cost categories: medical hospital, outpatient family therapy, medication visit, medication, doctor visit	
Mitchell [75]	Payer	USA	€3,221 Included cost categories: hospital care, healthcare provider, prescription medication	
O’Brien [60]	Payer	USA	€18,587 Included cost categories: inpatient cost	
Striegel-Moore [61]	Payer	USA	Female	€6,590
			Male	€2,993
			Included cost categories: inpatient treatment, outpatient treatment	
BN				
Crow [77]	Societal	USA	Face-to-face cognitive behavioral therapy	€2423
			Telemedicine cognitive behavioral therapy	€1488
			Included cost categories: evaluation and laboratory, cognitive behavioral therapy, subject travel, gasoline, therapist travel	
Crow [76]	Patient	USA	Total food cost	€4,735
			Binge/purge food cost	€1,357
			Objective binge eating food cost	€668
			Diet/laxative/diet pill cost	€201
Crow [31]	Payer	USA	Cognitive behavioral therapy	€3294
			Stepped care	€2824
			Included cost categories: cognitive behavioral therapy, self-help, medication, physician visit, emergency room, hospitalization, individual therapy, group therapy, medication management	
Koran [78]	Payer	USA	Cognitive behavioral therapy	€1761
			Med16	€888
			Med24	€1130
			Combi16	€2512
			Combi24	€2754
			Cost was calculated by multiplying the clinic’s professional fees by the number of visits and adding the cost of the patient’s expected medication and serum desipramine level	
Krauth [58]	Societal	Germany	€1460 Included cost categories: inpatient treatment, convalescence statutory health insurance, rehabilitation statutory pension insurance, inability to work, death	
Haas [73]	Hospital	Germany	€3386 Included cost categories: physicians, nursing, psychotherapists, dietician, art and music therapy, psychotherapy, drugs, medical products, services on demand, overhead costs (medical/non-medical infrastructure)	
Mitchell [75]	Payer	USA	€6279 Included cost categories: hospital care, healthcare provider, prescription medication	
O’Brien [60]	Payer	USA	€11,406 Included cost categories: inpatient care	
Pohjolainen [34]	Payer	Finland	€7756 Included cost categories: inpatient care, outpatient care, laboratory cost, radiology cost	
Striegel-Moore [61]	Payer	USA	Female	€3229
			Male	€4235
			Included cost categories: inpatient treatment, outpatient treatment	
Wang [79]	Payer	USA	€18,823 Included cost categories: school-based obesity prevention program	
BED				
Dickerson [68]	Payer	USA	BED	€2902
			Recurrent binge eating	€3137
			Included cost categories: weight- and eating disorder-related services, non-weight- and eating disorder-related mental health services, other provider-based services, medication services	
Grenon [39]	Payer	Canada	€1762 Included cost categories: family physician visits, medication use, diagnostic tests, health professionals’ visits, specialist visits, herbal remedies, other resources, outpatient visits, emergency department visits, and inpatient visits	

*AN* anorexia nervosa, *BED* binge eating disorder, *BN* bulimia nervosa, *EUR* euro

The healthcare costs of AN, BN, and BED were not compared in any of the included studies. However, 5 studies contrasted AN with BN [58, 60, 61, 73, 75] (Online Resource 2). In most of these studies, AN was found to be associated with considerably higher annual healthcare costs than BN (€5952 vs. €1460 [58], €18,587 vs. €11,406 [60], and €5445 vs. €3386 [73]). However, Mitchel et al. [75] estimated higher costs for BN (€6279) than AN (€3221). Striegel-Moore et al. [61] assessed the cost of EDs (i.e., AN, BN, and EDNOS) and compared these costs with the costs of other mental disorders (i.e., obsessive–compulsive disorder and schizophrenia). AN (€6590 for female, €2993 for male) and BN (€3229 for female, €4235 for male) patients had higher costs than obsessive–compulsive disorder patients (€2104 for female, €1965 for male) [61]. The healthcare costs for men with AN and BN were slightly less than the costs for men with schizophrenia (€5552); however, the costs were higher for women with schizophrenia compared to BN (€5259). The healthcare costs for patients with BN were comparable to the costs associated with the treatment of EDNOS (female €3229, male €4235 vs. female €3496, male €2360 [61], and €6279 vs. €6514 [75]). In one of the two studies that examined the costs of BED, the annual healthcare cost of overweight/obese BED women (€1,762) was reported to be 36.5 % higher than that of the age- and sex-matched national average [39]. Grenon et al. [39] found no association with BMI (after controlling for age) and the total healthcare costs in BED. However, this finding is in contrast to the study by Dickerson et al. [68] that showed a significant association with higher BMI and higher mental health medication and total medication costs in women with BED or recurrent BE.

In AN, premature death in the young patient population leads to significant indirect costs to society (63 % of the overall costs), mainly due to the loss of production [58]. Symptom-related costs for food are considerable expense factors for patients with BN. Crow et al. [76] found that costs associated with BE and purging in BN accounted for 32.7 % of the total food costs; the mean total annual food cost per BN patient was €4735. No study was identified for BED-related food costs.

## Discussion

A comprehensive review on the differences in the HRQoL and economic impact of AN, BN, and BED was missing from the literature. We analyzed 69 studies: data on HRQoL were reported in 41 studies (18 for AN, 17 for BN, and 18 for BED), on healthcare utilization in 20 studies (14 for AN, 12 for BN, and 8 for BED), and on healthcare costs in 17 studies (9 for AN, 11 for BN, and 2 for BED).

Generally, AN, BN, or BED was associated with significantly impaired HRQoL compared with the healthy population [18, 34, 37, 39, 40, 43]. Evidence also suggests that patients with EDs have impaired HRQoL compared to those with other psychiatric conditions [80]. Studies showed that obesity was associated with further deterioration of HRQoL of patients with BED [36, 37, 40, 43, 46]. In addition, other psychopathology factors (e.g., depression and anxiety) were associated with lower HRQoL of patients with AN, BN, and BED [19, 21, 24, 39, 43]. AN, BN, and BED may have differential effects on HRQoL compared with each other, depending mainly, but not exclusively, on the study design and the HRQoL domains investigated [15, 17, 22, 27]. The reviewed studies provided a range of results, at least partially due to the heterogeneity in the patient population and the research design. Consequently, the study results and conclusions seemed to be quite heterogeneous. It is difficult to draw a consistent conclusion on the relative effects of the assessed EDs on HRQoL, which may be because AN, BN, and BED share certain psychopathology symptoms (e.g., loss of control of eating appears both in BN and BED). In a recently published meta-analysis, Winkler et al. [81] could also not identify any specific differences between the HRQoL effects of different ED diagnostic groups. The HRQoL of the siblings and caregivers of AN, BN, and BED patients was also found to be impaired [49–53].

The economic impacts of EDs has been receiving increasing research attention in recent years [82–84]; however, a comparative review on the economic burden of AN, BN, and BED was lacking in the literature. Based on the reviewed studies, AN, BN, and BED are associated with increased healthcare utilization and higher healthcare costs. The evaluated EDs are associated with a high rate of hospitalization, outpatient care, and emergency department visits [3, 54, 58–62, 64–66, 69]. However, inpatient care seems to be more common in AN [54, 58, 60, 61, 64], which could primarily be due the severity of the condition. It was also notable that patients rarely receive specific treatment for their ED and mostly received treatment for general mental health, comorbid psychiatric symptoms, and/or weight loss [2, 3, 59, 66]. Improvement in diagnosis, reporting/coding and management of AN, BN, and BED may improve treatment for these patients.

The cost estimates varied considerably for AN, BN, and BED, as well as within particular disorders. Several differences in the design and analysis of the cost of illness in the studies reduced the comparability of the cost data. The annual direct healthcare costs of AN and BN appeared to be similar to those of other major psychiatric conditions [61]. Compared with BN, AN was associated with higher healthcare costs in most assessed studies [58, 60, 72]. Healthcare cost data showed variation across countries; however, an investigation of the factors behind the cross-country differences was beyond the scope of this review (differences in treatment patterns and evaluated cost factors as well as national characteristics of the healthcare systems are likely to be key determinants).

Compared to BN, obesity appears to be more common in BED [2]. Due to the limited number of studies, it is not possible to definitively separate burden specific to BED from those associated with obesity. Namely, a higher level of BMI decreases the physical HRQoL of individuals (e.g., worst physical functioning) [85]. Nevertheless, BED is distinct from obesity; there is a unique psychopathology and medical risk profile associated with BED [86]. Patients with BED have greater functional impairment, lower HRQoL, and more subjective distress compared to weight-matched obese individuals without BED [36, 37, 40, 44, 46]. This suggests that at least part of the burden of BED is unique to the disorder and provides a rationale for not only treating weight issues, but the disorder itself to fully address the burden of BED. However, obesity may have severe health consequences over time (e.g., diabetes and hypertension), which may have an impact on the long-term burden of BED. Although BED is the most prevalent ED, there is scarcity of information on the economic burden of BED; those studies that do report the economic burden of BED, most reported it in combination with BN or overall EDs (only 2 studies reported healthcare cost data for BED [39, 68]). Further research is needed on this topic, because data on the long-term health and economic impact of BED are essential to better establish the burden of illness in this patient population.

Our findings should be considered in light of the following limitations. The current systematic literature review involved searching only studies that were published in English and indexed in the selected databases. Despite the broad database selection, the inclusion of unpublished material and studies from other resources was precluded. As a further effort, we screened the references of the included articles for additional eligible studies. No formal quality assessment of the identified publications was performed, because of the large heterogeneity in the study types of the included studies.

In summary, AN, BN, and BED markedly impair HRQoL and are associated with increased healthcare use and costs compared with healthy individuals. The HRQoL impairment of AN, BN, and BED patients was found to be similar; however, healthcare utilization (especially, inpatient care) and costs were reported to be higher for AN patients than for BN and BED patients. Compared to BN, obesity appears to be more common in patients with BED, which may contribute to significant impairment of their HRQoL and higher healthcare use, especially in the long term. Thus, the burden of BED should be examined separately from that of BN. The limited evidence indicates that further research is warranted to better understand the differences between the HRQoL and economic burdens of BED in contrast to AN and BN in the long term.

## Electronic supplementary material

Below is the link to the electronic supplementary material.

Supplementary material 1 (DOCX 36 kb)

Supplementary material 2 (DOCX 87 kb)

Supplementary material 3 (DOCX 94 kb)

Supplementary material 4 (DOCX 53 kb)

Supplementary material 5 (DOCX 55 kb)
