# Supplementary material for: A systematic review of the health-related quality of life and economic burdens of anorexia nervosa, bulimia nervosa, and binge eating disorder
Source: Eat Weight Disord. 2016 Mar 4;21(3):353–64. doi: 10.1007/s40519-016-0264-x (PMC5010619; doi:10.1007/s40519-016-0264-x)
Supplement: Supplementary file 2 — Supplementary material 2 (DOCX 87 kb) [file 40519_2016_264_MOESM2_ESM.docx]

# **Online Resource 2.** General characteristics of the included studies

| **First author, year** | **Study characteristic** | | | | | **Characteristics of study samples** | | | | |
| --- | --- | --- | --- | --- | --- | --- | --- | --- | --- | --- |
|  | **Country** | **Study design** | **Study year** | **Included eating disorders** | **Diagnostic criteria** | **Sample** | **Sample size** | **Sample age** range/mean(SD) | **Percentage of female** | **BMI** range/mean(SD) |
| Abraham, 2006 [14] | Australia | LS (12 months) | - | AN, BN, EDNOS | DSM-IV | AN | 74+34 | Sample 1: 21.8(7.8) Sample 2: 19.3(5.9) | 100% | Sample 1: 19.4(4.3) Sample2: 17.7(3.0) |
|  |  |  |  |  |  | BN | 33+10 |  |  |  |
|  |  |  |  |  |  | EDNOS | 80+21 |  |  |  |
|  |  |  |  |  |  | No diagnosis | 54 |  |  |  |
| Abraham, 2011 [13] | Australia | CS | - | AN, BN, EDNOS | DSM-IV | AN | 71 | 24.0(6.0) | 100% | 15.5(1.5) |
|  |  |  |  |  |  | BN | 29 | 25.0(6.0) | 100% | 22.6(3.4) |
|  |  |  |  |  |  | EDNOS | 60 | 25.0(7.0) | 100% | 19.6(2.4) |
| Areemit, 2010 [49] | Canada | CS | 2008 | Sibling of AN and EDNOS adolescents | - | Sibling | 20 | 13.7(2.1) | 70% |  |
|  |  |  |  |  |  | Patients | 17 | 14.5(1.8) | 88% |  |
| Bamford, 2010 [15] | UK | CS | - | AN, BN, EDNOS | DSM-IV | AN-R | 56 | 26.7(7.7) | 95% | 13.9(1.6) |
|  |  |  |  |  |  | AN-P | 24 |  |  | 14.8(1.4) |
|  |  |  |  |  |  | BN | 40 |  |  | 22.4(2.6) |
|  |  |  |  |  |  | EDNOS | 36 |  |  | 22.0(5.1) |
|  |  |  |  |  |  | TOTAL | 156 |  |  | 18.1(5.0) |
| Byford, 2007 [70] | UK | CEA | 2000-2003 | AN | DSM-IV | AN | 167 |  |  |  |
| Calderon, 2007 [54] | US | DA | 2000-2004 | AN, BN, EDNOS | ICD-9 | AN | 1,208 | 15.1(2.3) | 92% |  |
|  |  |  |  |  |  | BN | 255 | 16.7(2.0) | 98% |  |
|  |  |  |  |  |  | EDNOS | 250 | 14.8(2.6) | 84% |  |
| Cassin, 2008 [35] | Canada | LS (16 weeks) | 2004-2005 | BED | DSM-IV | BED, AMI | 54 | 42.5(12.7) | 100% | 33.2(7.8) |
|  |  |  |  |  |  | BED, control | 54 |  |  |  |
| Crow, 2013 [31] | US | LS (4 months) | 2005 | BN | DSM-IV | BN, CBT | 147 | 29.5(8.0) | 100% | 23.4(4.5) |
|  |  |  |  |  |  | BN, Stepped Care | 146 | 29.8(9.8) | 100% | 23.5(5.3) |
| Crow, 2009 [77] | US | CEA | - | BN | DSM-IV | BN, FTF-CBT | 66 | 29.6(10.9) | 97% | 23.3(5.0) |
|  |  |  |  |  |  | BN, TV-CBT | 62 | 28.4(10.4) | 100% | 23.5(5.4) |
| Crow, 2009 [76] | US | CS | 2004-2007 | BN | - | BN | 10 | 26.6(6.2) | 100% |  |
| Crow, 2004 [71] | US | CEA | - | AN | - |  |  |  |  |  |
| De La Rie, 2005 [50] | The Netherlands | CS | - | Caregivers of patients with AN, BN, BED, EDNOS | - | caregivers | 40 | 46.0(10.7) | 63% |  |
|  |  |  |  |  |  | patients | 40 |  |  |  |
| del Valle, 2010 [16] | Spain | LS (12 weeks) | 2007 | AN-R | - | Training group | 11 | 14.7(0.6) | 91% | 18.7(1.7) |
|  |  |  |  |  |  | Control group | 11 | 14.2(1.2) | 91% | 18.2(1.5) |
| de Zwaan, 2002 [36] | US | CS | - | BED | DSM-IV | TOTAL, Preoperative | 110 | 17-61/40.3 | 87% | 32.1-57.2/43.8 |
|  |  |  |  |  |  | TOTAL, Postoperative | 78 | 31-77/32.8 | 83% | 22.7-49.5/32.8 |
| de Zwaan, 2002 [37] | US | CS | 1999-2001 | BED | DSM-IV | BED | 19 | 41.3(9.0) | 100% | 48.0(7.9) |
|  |  |  |  |  |  | TOTAL | 110 | 19-62/39.2 | 87% | 35.4-86.9/48.4 |
| Dickerson, 2011 [68] | US | CS | - | BED, RBE | DSM-IV | BED | 50 | 36.4(7.3) | 100% | 33.4(6.1) |
|  |  |  |  |  |  | RBE | 50 | 38.6(8.3) | 100% | 30.9(5.7) |
| Doll, 2005 [17] | UK | CS | 1996 | AN, BN, BED | DSM-IV | TOTAL | 1,439 | 23.4(6.8) | 63% | 22.6(3.4) |
| Faulconbridge, 2012 [38] | US | LS (12 months) | - | BED | DSM-V | BED, bariatric surgery | 36 | 47.0(1.6) | 73% | 48.9(1.1) |
|  |  |  |  |  |  | BED, lifestyle modification | 49 | 43.8(1.4) | 80% | 44.3(0.7) |
| Fox, 2009 [18] | UK | CS | - | AN | - | AN | 43 | 32.7(9.6) | 100% |  |
|  |  |  |  |  |  | Control | 56 | 28.5(8.6) |  |  |
| George, 1987 [55] | US | DA | 1958-1962, 1968-1972, 1978-1982 | AN | - | AN | 76 |  |  |  |
| Gonzalez, 2012 [51] | Spain | CS |  | Caregivers of patients with AN, BN, EDNOS | DSM-IV | Caregivers | 143 | 49.9(11.2) | 55% |  |
|  |  |  |  |  |  | Patients | 84 | 26.5(9.5) | 100% |  |
| Gonzalez-Pinto, 2004 [19] | Spain | CS | 1999-2000 | AN | DSM-IV | AN | 47 | 20.0(5.5) | 83% | 16.6(1.7) |
| Grenon, 2010 [39] | Canada | CS | - | BED | DSM-IV | BED | 105 | 44.3(11.8) | 100% | 38.2(6.8) |
| Grigoriadis, 2001 [56] | Canada | LS (24 weeks) |  | AN |  | AN | 24 | 31.0(9.2) | 100% | 19.9(4.0) |
| Haas, 2012 [72] | Germany | DA | 2005-2009 | AN | ICD-10 | AN | 127 | 25.7(7.6) | 97% | 13.8(1.9) |
| Haas, 2012 [73] | Germany | DA | 2006-2009 | AN, BN | ICD-10 | AN | 101 | 26.4(7.4) | 96% | 13.7(2.0) |
|  |  |  |  |  |  | BN | 95 | 25.0(5.6) | 100% | 20.7(5.3) |
|  |  |  |  |  |  | Obese | 60 | 43.4(13.7) | 69% | 46.5(8.3) |
| Hsu, 2002 [40] | US | CS | - | BED | DSM-IV | BED | 9 | 41.1(7.4) | 78% | 54.2(11.8) |
|  |  |  |  |  |  | TOTAL | 37 |  | 84% |  |
| Jager, 2004 [67] | Germany | LS (8 years) | - | BN | DSM-III | BN | 80 | 31.7(4.1) | 100% |  |
| Kalisvaart, 2007 [57] | US | DA | 2001-2003 | AN, EDNOS | - | TOTAL | 39 | 16.1(1.9) |  |  |
| Keilen, 1994 [20] | UK | CS | 1991-1992 | AN, BN | DSM-III | AN | 62 |  | 92% |  |
|  |  |  |  |  |  | BN | 80 |  | 100% |  |
|  |  |  |  |  |  | Control | 95 |  | 100% |  |
| Kessler, 2013 [2] | Colombia, Brazil, Mexico, Romania, Belgium, France, Germany, Italy, The Netherlands, New Zealand, Northern Ireland, Portugal, Spain, US | CS | - | BN, BED | DSM-IV | TOTAL | 24,124 | 18+ |  |  |
| Kolotkin, 2004 [41] | US | CS | - | BED | DSM | BED | 95 | 45.0(12.6) | 76% | 42.0(10.4) |
|  |  |  |  |  |  | Non-BED | 435 | 49.7(14.3) | 56% | 38.5(10.3) |
| Koran, 1995 [78] | US | CEA | - | BN | DSM-III | BN | 71 | 18-65 | 100% |  |
| Krauth, 2002 [58] | Germany | Cost study | - | BN | ICD-10 |  |  |  |  |  |
| Latner, 2008 [21] | New Zealand | CS | - | AN, BN, BED, EDNOS | DSM-IV | AN | 11 | 26.3(8.9) | 100% | 21.6(7.3) |
|  |  |  |  |  |  | BN | 5 |  |  |  |
|  |  |  |  |  |  | BED | 3 |  |  |  |
|  |  |  |  |  |  | EDNOS | 30 |  |  |  |
| Lock, 2008 [74] | US | CEA | - | AN | - | AN | 86 | 15.2 |  |  |
| Marchesini, 2002 [42] | Italy | LS (5 months) |  | BED | - | CBT | 92 | 43 | 84% | 36.7(5.1) |
|  |  |  |  |  |  | Control | 76 | 43 | 88% | 35.1(6.5) |
| Marques, 2011 [65] | US | database analysis | - | AN, BN, BED | DSM-IV |  |  |  |  |  |
| Martin, 2011 [52] | Spain | CS | - | Caregivers of patients with: AN, BN, ED | DSM-IV | Caregivers | 246 | 47.9(12.4) | 53% |  |
|  |  |  |  |  |  | Patients | 145 | 25.6(8.9) | 99% |  |
| Masheb, 2004 [43] | US | CS | - | BED | DSM-IV | BED | 94 | 44.9(8.3) | 78% | 35.2(8.1) |
| Mitchell, 2009 [75] | USA | DA | 1999-2005 | AN, BN, EDNOS, no ED | - | Post ED | 167 | 31.4 |  |  |
|  |  |  |  |  |  | Pre ED | 155 | 32.2 |  |  |
|  |  |  |  |  |  | Depressed | 224 | 32.2 |  |  |
|  |  |  |  |  |  | No ED | 6,866 | 31.7 |  |  |
| Mond, 2010 [32] | Australia | CS | - | BN | DSM-IV | Objective bulimic episodes | 37 | 29.4(6.4) | 100% | 28.9(7.8) |
|  |  |  |  |  |  | Subjective bulimic episodes | 52 | 28.6(6.5) |  | 25.7(5.2) |
|  |  |  |  |  |  | Objective and subjective bulimic episodes | 13 | 27.2(7.9) |  | 31.1(8.4) |
| Mond, 2007 [59] | Australia | CS | - | AN, BN, BED, EDNOS | DSM-IV | BED | 31 | 28.9(6.6) | 100% | 27.2(7.1) |
|  |  |  |  |  |  | AN | 18 |  |  |  |
|  |  |  |  |  |  | BN | 51 |  |  |  |
|  |  |  |  |  |  | EDNOS | 59 |  |  |  |
|  |  |  |  |  |  | Control | 5096 | 30.3(7.2) |  | 24.4(5.2) |
| Mond, 2005 [22] | Australia | CS | 2001-2002 | AN, BN, BED | DSM-IV | BED | 10 | 34.3(7.4) | 100% | 41.2(11.7) |
|  |  |  |  |  |  | BN | 40 | 23.5(6.3) | 100% | 24.0(4.4) |
|  |  |  |  |  |  | AN-P | 15 | 25.5(9.8) | 100% | 18.3(2.1) |
|  |  |  |  |  |  | AN-R | 19 | 19.3(4.2) | 100% | 17.2(1.3) |
|  |  |  |  |  |  | Control | 495 | 33.5(8.9) | 100% | 24.9(6.5) |
| Munoz, 2009 [23] | Spain | LS (12 months) | - | AN, BN, EDNOS | DSM-IV | AN | 61 | 26.8 | 96.6% | 21.4 |
|  |  |  |  |  |  | BN | 47 |  |  |  |
|  |  |  |  |  |  | EDNOS | 245 |  |  |  |
| Nickel, 2005 [33] | Germany | LS (10 weeks) | 2004 | BN | DSM-IV | BN, topiramate group | 30 | 21.5(3.1) | 100% | 22.7 |
|  |  |  |  |  |  | BN, placebo group | 30 | 21.1(2.6) | 100% | 22.7 |
| O'Brien, 2003 [60] | US | DA | - | AN, BN | ICD-9 | AN | 641 | 25 | 96% |  |
|  |  |  |  |  |  | BN | 326 | 27 | 97% |  |
| Padierna, 2013 [53] | Spain | CS | - | Caregivers of patients with AN, BN | DSM-IV | Caregivers | 246 | 47.9(12.4) |  |  |
|  |  |  |  |  |  | Patients | 145 | 25.7(8.9) | 99% |  |
| Padierna, 2000 [24] | Spain | CS | - | AN, BN, BED | DSM-IV | BED | 17 |  |  |  |
|  |  |  |  |  |  | AN-R | 56 |  |  |  |
|  |  |  |  |  |  | AN-P | 60 |  |  |  |
|  |  |  |  |  |  | BN | 64 |  |  |  |
|  |  |  |  |  |  | TOTAL | 197 | 23.4(6.7) | 98% |  |
| Perez, 2012 [44] | US | DA | - | BED | DSM-IV | BED | nonobese: 124 obese: 126 |  |  | nonobese: 25.3(3.2) obese: 36.3(5.2) |
|  |  |  |  |  |  | TOTAL | 16,898 |  | 57% |  |
| Pohjolainen, 2010 [34] | Finland | LS (6 months) | 2002-2003 | BN | ICD-10 | TOTAL | 72 | 25.0(6.0) | 100% | 22.0(3.9) |
| Preti, 2009 [3] | Belgium, France, Germany, Italy, The Netherlands, Spain | CS | 2001-2003 | AN, BN, BED | DSM-IV | TOTAL | 21,425 | 18+ | 52% |  |
| Ricca, 2009 [45] | Italy | CS | 2004-2007 | BED | DSM-IV | BED  TOTAL | 105  438 | 44.9(12.7) | 88% | 38.1(7.4) |
| Rie, 2005 [25] | The Netherlands | CS | - | BN, AN, EDNOS, former ED | DSM-IV | AN | 44 | 26.3(9.1) | 100% | 15.6(1.5) |
|  |  |  |  |  |  | BN | 43 | 29.0(7.8) | 95% | 21.6(3.5) |
|  |  |  |  |  |  | EDNOS | 69 | 29.4(9.1) | 99% | 21.0(5.9) |
|  |  |  |  |  |  | Former ED | 148 | 28.7(8.9) | 97% | 22.3(5.0) |
| Rieger, 2005 [46] | US | CS | - | BED | DSM-IV | BED | 56 | 42.0(10.0) | 89% | 36.6(5.1) |
|  |  |  |  |  |  | Non-BED | 62 | 36.6(5.1) | 81% | 37.0(4.9) |
| Silveira, 2005 [47] | Brazil | LS (12 weeks) | - | BED | DSM-IV | BED | 9 | 33.3(10.8) | 100% | 36.5(4.5) |
| Striegel-Moore, 2008 [62] | USA | DA | - | AN, BN, EDNOS | - | AN | 28 | 28.1(8.5) | 100% |  |
|  |  |  |  |  |  | BN | 77 | 31.5(9.8) | 100% |  |
|  |  |  |  |  |  | EDNOS | 99 | 30.7(11.0) | 100% |  |
| Striegel-Moore, 2004 [69] | USA | CS | - | BED | DSM-IV | BED | 162 |  | 100% |  |
|  |  |  |  |  |  | TOTAL | 518 |  | 100% |  |
| Striegel-Moore, 2000 [61] | US | DA | 1995 | AN, BN, EDNOS | ICD-9 | AN, female | 517 | 24.8(12.5) | 100% |  |
|  |  |  |  |  |  | AN, male | 49 | 28.6(17.6) | 0% |  |
|  |  |  |  |  |  | BN, female | 725 | 27.9(10.7) | 100% |  |
|  |  |  |  |  |  | BN, male | 41 | 32.0(15.8) | 0% |  |
|  |  |  |  |  |  | EDNOS, female | 756 | 30.4(13.4) | 100% |  |
|  |  |  |  |  |  | EDNOS, male | 176 | 31.6(18.6) | 0% |  |
| Swanson, 2011 [66] | USA | CS | - | AN, BN, BED | DSM-IV | TOTAL | 10123 | 13-18 |  |  |
| Thien, 2000 [26] | Canada | LS | 1997 | AN | DSM-IV | AN, Graded Exercise Program | 5 | 29.0(4.4) | 100% | 20.3(1.8) |
|  |  |  |  |  |  | AN, control | 7 | 36.1(7.9) | 86% | 17.2(1.6) |
| Turner, 2010 [27] | UK | CS | 2004-2005 | AN, BN, EDNOS | DSM-IV | AN | 14 | 23.7(5.6) | 100% | 15.4 (1.2) |
|  |  |  |  |  |  | BN | 66 | 26.3(7.3) | 99% | 23.9 (5.6) |
|  |  |  |  |  |  | EDNOS | 98 | 27.3(8.4) | 96% | 21.6 (6.4) |
| Wales, 2013 [63] | UK | DA |  | AN, EDNOS | DSM-IV | AN-R | 94 |  |  | 15.2(1.4) |
|  |  |  |  |  |  | AN-P | 24 |  |  | 16.1(1.5) |
|  |  |  |  |  |  | EDNOS | 26 |  |  | 16.0(1.4) |
| Walsh, 2006 [28] | US | RCT | 2000-2005 | AN | DSM-IV | AN, fluoxetine | 49 | 22.4(4.5) | 100% | 19.1(2.1) |
|  |  |  |  |  |  | AN, placebo | 44 | 24.2(4.5) |  | 18.4(1.6) |
| Wang, 2011 [79] | US | CEA |  | BN |  |  |  |  |  |  |
| Watson, 2013 [30] | Australia | CS | 2004-2011 | AN, BN, subjective BN | DSM-5 | AN-R | 45 | 25.0(5.9) | 98% | 16.0(1.4) |
|  |  |  |  |  |  | AN-P | 24 |  |  | 16.1(1.4) |
|  |  |  |  |  |  | BN | 112 |  |  | 23.5(3.4) |
|  |  |  |  |  |  | Subjective BN | 28 |  |  | 21.1(2.5) |
| Watson, 2012 [29] | Australia | LS | 2004-2009 | AN, BN, EDNOS | DSM-IV | AN | 34 | 25.2(9.5) | 97% | 15.9(1.4) |
|  |  |  |  |  |  | BN | 87 | 26.8(7.9) | 100% | 22.7(3.2) |
|  |  |  |  |  |  | EDNOS | 75 | 25.5(8.5) | 99% | 20.7(3.1) |
|  |  |  |  |  |  | All EDs | 196 | 26.0(8.4) | 99% | 20.8(3.8) |
| Wilfley, 2008 [48] | - | LS (24 weeks) | - | BED | DSM-IV | BED, sibutramine | 152 | 41.8(9.7) | 90% | 35.5(5.8) |
|  |  |  |  |  |  | BED, placebo | 152 | 42.1(9.9) | 90% | 36.3(5.5) |
| Williamson, 2001 [64] | USA | CEA | - | AN, BN | DSM-IV | AN, inpatient | 20 | 23.0(8.2) | 100% | 17.0(1.8) |
|  |  |  |  |  |  | AN, day patient | 16 | 22.5(9.4) | 100% | 18.3(1.8) |
|  |  |  |  |  |  | BN, inpatient | 8 | 28.0(10.6) | 100% | 21.9(3.9) |
|  |  |  |  |  |  | BN, day patient | 7 | 25.6(6.0) | 100% | 20.6(3.3) |

AMI: adapted motivational interview, AN: anorexia nervosa, AN-R: anorexia nervosa restricting type, AN-P: anorexia nervosa purging type, BED: binge eating disorder, BN: bulimia nervosa, CBT: cognitive behavioral therapy, CEA: cost-effectiveness analysis, CS: cross-sectional study, DA: database analysis, DSM: Diagnostic and Statistical Manual of Mental Disorders, EDNOS: eating disorder not otherwise specified, FTF-CBT: face-to-face cognitive behavioral therapy, ICD: International Classification of Diseases, LS: longitudinal study, RBE: recurrent binge eating, TV-CBT: telemedicine cognitive behavioral therapy, UK: United Kingdom, US: United States
