# Supplementary material for: A systematic review of the health-related quality of life and economic burdens of anorexia nervosa, bulimia nervosa, and binge eating disorder
Source: Eat Weight Disord. 2016 Mar 4;21(3):353–64. doi: 10.1007/s40519-016-0264-x (PMC5010619; doi:10.1007/s40519-016-0264-x)
Supplement: Supplementary file 3 — Supplementary material 3 (DOCX 94 kb) [file 40519_2016_264_MOESM3_ESM.docx]

# **Online Resource 3.** Selected health-related quality of life burden data on AN, BN, and BED

| **First author, year** | **Included eating disorders** | **Sample size** | **Instrument** | **Reported HRQoL scores**  (as mean (SD), or specified) |
| --- | --- | --- | --- | --- |
| Abraham, 2006 [14] | AN, BN, EDNOS | AN: 74+34  BN: 33+10  EDNOS: 80+21 | SF-12,  QOL-ED | No ED diagnosis-specific SF-12 or QOL-ED scores were presented. |
| Abraham, 2011 [13] | AN, BN, EDNOS | AN: 71  BN: 29  EDNOS: 60 | QOL-ED | \| **QOL ED scale scores by diagnostic groups** \| \| \| \| \| --- \| --- \| --- \| --- \| \|  \| **AN** \| **BN** \| **EDNOS** \| \| Global \| 17.1 (3.0) \| 15.7 (2.9) \| 15.7 (3.4) \| \| Body weight \| 2.79 (1.13) \| 0.34 (0.61) \| 0.95 (1.03) \| \| Eating behavior \| 2.20 (0.79) \| 3.07 (0.59) \| 2.50 (0.77) \| \| ED \| 3.34 (0.88) \| 3.72 (0.53) \| 3.53 (0.77) \| \| Psychological \| 3.21 (0.93) \| 3.24 (0.79) \| 3.33 (0.82) \| \| Daily living \| 3.20 (0.91) \| 3.36 (0.85) \| 3.09 (1.0) \| \| Acute medical \| 2.34 (1.33) \| 1.93 (1.53) \| 2.30 (1.41) \| |
| Bamford, 2010 [15] | AN, BN, EDNOS | AN-R: 56  AN-P: 24  BN: 40  EDNOS: 36 | EDQoL | \| **EDQoL scales scores by diagnostic groups** \| \| \| \| \| \| --- \| --- \| --- \| --- \| --- \| \|  \| **AN-R** \| **AN-P** \| **BN** \| **EDNOS** \| \| Psychological \| 2.62 (0.66) \| 2.54 (0.99) \| 2.95 (0.80) \| 3.11 (0.66) \| \| Physical/cognitive \| 1.92 (0.70) \| 1.83 (0.91) \| 2.22 (0.95) \| 2.36 (0.91) \| \| Financial \| 0.54 (0.76) \| 0.67 (0.93) \| 0.68 (1.00) \| 0.79 (1.08) \| \| Work/school \| 0.82 (0.99) \| 0.79 (0.96) \| 1.21 (1.27) \| 1.38 (1.44) \| \| Global \| 1.65(0.68) \| 1.54 (0.71) \| 1.84 (0.77) \| 2.07 (0.81) \| |
| Cassin, 2008 [35] | BED | BED, adapted motivational interviewing: 54  BED, control: 54 | ESWLS | \| **ESWLS scale scores by study groups** \| \| \| \| \| \| --- \| --- \| --- \| --- \| --- \| \|  \| **Baseline** \| \| **16 weeks follow-up** \| \| \|  \| **Control** \| **Adapted motivational interviewing** \| **Control** \| **Adapted motivational interviewing** \| \| General life \| 17.1 (8.0) \| 16.5 (7.8) \| 19.6 (8.4) \| 21.9 (8.4) \| \| Social life \| 15.8 (8.5) \| 14.1 (8.0) \| 18.5 (10.3) \| 18.5 (8.9) \| \| Sex life \| 13.5 (8.7) \| 12.0 (8.0) \| 15.3 (9.1) \| 15.9 (9.3) \| \| Self \| 16.0 (6.9) \| 14.1 (6.5) \| 19.4 (7.5) \| 20.4 (8.3) \| \| Physical appearance \| 8.7 (4.9) \| 7.1 (3.9) \| 10.2 (6.0) \| 10.6 (6.6) \| \| Family \| 18.4 (10.1) \| 19.5 (8.8) \| 20.9 (9.3) \| 23.8 (8.6) \| \| Relationships \| 19.2 (9.3) \| 17.5 (10.1) \| 20.6 (9.2) \| 22.1 (9.2) \| |
| Crow, 2013 [31] | BN | BN, CBT: 147  BN, stepped care: 146 | Quality of Well-Being Scale - Self Administered | \| **Quality of well-being scale – self-administered version total score by study groups** \| \| \| \| \| \| --- \| --- \| --- \| --- \| --- \| \|  \| **CBT** \| \| **Stepped care** \| \| \|  \| **Baseline** \| **Week 62** \| **Baseline** \| **Week 62** \| \| Total score \| 0.598 (0.101) \| 0.699 (0.152) \| 0.597 (0.106) \| 0.685 (0.151) \| |
| de Zwaan, 2002 [36] | BED | Preoperative group: 110 (BED: 19)  Postoperative group: 78 (BED: 9) | SF-36 | \| **SF-36 scale and summary scores by study groups** \| \| \| \| \| --- \| --- \| --- \| --- \| \|  \| **Control group - Preoperative** \| **Postoperative group** \| **General US**  **population** \| \| Physical functioning \| 41.6 (18.8) \| 85.7 (19.7) \| 84.2 (23.3) \| \| Role - physical \| 41.3 (36.2) \| 76.6 (34.9) \| 80.9 (34.0) \| \| Bodily pain \| 45.6 (21.0) \| 62.1 (28.1) \| 75.2 (23.7) \| \| General health \| 41.4 (20.1) \| 67.2 (26.0) \| 71.9 (20.3) \| \| Vitality \| 29.9 (19.4) \| 56.9 (21.7) \| 60.9 (20.9) \| \| Social functioning \| 60.4 (24.9) \| 87.8 (22.5) \| 83.3 (22.7) \| \| Role - emotional \| 74.9 (34.4) \| 92.3 (22.1) \| 81.3 (33.0) \| \| Mental health \| 70.5 (17.4) \| 74.6 (18.9) \| 74.7 (18.1) \| \| PCS \| 30.6 (8.9) \| 46.6 (10.1) \| 50.0 (10.0) \| \| MCS \| 48.9 (10.1) \| 52.1 (8.4) \| 50.0 (10.0) \|      \| **SF-36 scale scores in BED versus non-BED patients in the postoperative group** \| \| \| \| --- \| --- \| --- \| \|  \| **BED** (n=9) \| **non-BED** (n=69) \| \| Physical functioning \| 82.8 (16.4) \| 86.1 (20.1) \| \| Role - physical \| 61.1 (28.3) \| 78.6 (35.4) \| \| Bodily pain \| 55.3 (26.0) \| 62.9 (28.4) \| \| General health \| 42.4 (30.9) \| 70.4 (23.7) \| \| Vitality \| 41.7 (25.9) \| 58.9 (20.5) \| \| Social functioning \| 72.4 (31.5) \| 89.8 (20.5) \| \| Role - emotional \| 92.6 (22.3) \| 92.3 (22.3) \| \| Mental health \| 59.1 (24.9) \| 76.6 (17.2) \| \| PCS \| 42.2 (8.9) \| 47.2 (10.2) \| \| MCS \| 45.7 (10.4) \| 52.8 (7.8) \| |
| de Zwaan, 2002 [37] | BED | BED: 19  TOTAL: 110 | SF-36,  IWQOL-Lite | \| **SF-36 scale and summary scores by study groups** \| \| \| \| \| --- \| --- \| --- \| --- \| \|  \| **No BED** \| **BED** \| **US norms** \| \| Physical functioning \| 42.6 (19.7) \| 36.7 (13.3) \| 84.2 (23.3) \| \| Role–physical \| 45.0 (37.6) \| 23.5 (21.2) \| 80.9 (34.0) \| \| Bodily pain \| 46.9 (22.0) \| 39.1 (13.9) \| 75.2 (23.7) \| \| General health \| 41.9 (20.8) \| 39.1 (16.7) \| 71.9 (20.3) \| \| Vitality \| 32.1 (19.8) \| 19.4 (13.3) \| 60.9 (20.9) \| \| Social functioning \| 62.3 (26.2) \| 51.4 (14.9) \| 83.3 (22.7) \| \| Role–emotional \| 79.5 (30.7) \| 53.6 (43.2) \| 81.3 (33.0) \| \| Mental health \| 70.9 (18.2) \| 68.4 (13.1) \| 74.7 (18.1) \| \| PCS \| 31.1 (9.2) \| 28.1 (6.8) \| 50.0 (10.0) \| \| MCS \| 49.8 (10.1) \| 44.7 (9.5) \| 50.0 (10.0) \|  \| **IWQOL scale and total scores by study groups** \| \| \| \| \| --- \| --- \| --- \| --- \| \|  \| **No BED** \| **BED** \| **Community sample** \| \| Physical function \| 36.2 (20.2) \| 26.8 (13.6) \| 89.9 (14.9) \| \| Self-esteem \| 44.5 (22.9) \| 29.6 (16.1) \| 87.5 (19.4) \| \| Sexual life \| 55.3 (29.6) \| 38.7 (20.6) \| 95.1 (13.0) \| \| Public distress \| 49.4 (22.9) \| 42.2 (22.7) \| 96.5 (10.9) \| \| Work \| 67.6 (25.6) \| 49.3 (14.3) \| 95.4 (11.5) \| \| Total score \| 46.7 (17.6) \| 34.0 (9.9) \| 91.8 (12.0) \| |
| del Valle, 2010 [16] | AN | AN, training group: 11  AN, control group: 11 | SF-36 | \| **SF-36 scale and summary scores by study groups** \| \| \| \| \| --- \| --- \| --- \| --- \| \| **Scales** \| **Group** \| **Pre** \| **Post** \| \| Physical function \| Control \| 83.2 (17.6) \| 88.9 (19.8) \| \| Training \| 93.2 (7.5) \| 90.4 (11.9) \| \| Physical role \| Control \| 45.6 (39.8) \| 73.1 (33.1) \| \| Training \| 67.6 (21.1) \| 83.5 (22.7) \| \| Pain \| Control \| 59.7 (26.5) \| 73.9 (18.5) \| \| Training \| 95.6 (7.4) \| 91.3 (14.9) \| \| General health \| Control \| 52.2 (21.0) \| 56.2 (20.4) \| \| Training \| 70.7 (14.5) \| 72.3 (20.4) \| \| Vitality \| Control \| 51.3 (33.5) \| 57.0 (20.7) \| \| Training \| 76.7 (15.3) \| 72.2 (23.3) \| \| Social function \| Control \| 49.4 (28.3) \| 67.5 (19.7) \| \| Training \| 66.1 (18.7) \| 82.9 (18.7) \| \| Emotional role \| Control \| 56.8 (32.0) \| 68.3 (28.0) \| \| Training \| 72.5 (24.3) \| 88.6 (17.6) \| \| Mental health \| Control \| 40.2 (25.2) \| 46.0 (18.9) \| \| Training \| 73.2 (14.5) \| 66.8 (19.3) \| \| PCS \| Control \| 44.3 (11.5) \| 53.5 (8.1) \| \| Training \| 58.8 (14.0) \| 55.4 (6.1) \| \| MCS \| Control \| 35.1 (16.8) \| 39.6 (19.9) \| \| Training \| 44.2 (9.4) \| 47.9 (9.9) \| |
| Doll, 2005 [17] | AN, BN, BED, no ED, | AN: 7  BN:54  BED: 22  No ED: 1,356 | SF-36 | \| **SF-36 scale and summary scores by diagnostic groups** \| \| \| \| \| \| --- \| --- \| --- \| --- \| --- \| \|  \| **AN** \| **BN** \| **BED** \| **No ED** \| \| Physical function \| 88.92 (11.40) \| 90.62 (11.90) \| 87.23 (11.55) \| 89.86 (20.88) \| \| Role physical \| 73.75 (34.42) \| 67.71 (35.99) \| 52.49 (34.92) \| 73.38 (63.08) \| \| Role emotional \| 49.38 (42.03) \| 35.47 (43.95) \| 23.78 (42.57) \| 58.33 (77.20) \| \| Social functioning \| 72.37 (20.38) \| 70.51 (21.32) \| 72.28 (20.68) \| 78.89 (37.28) \| \| Mental health \| 52.57 (17.27) \| 51.84 (18.05) \| 52.78 (17.51) \| 64.54 (32.03) \| \| Vitality \| 41.63 (19.15) \| 42.85 (20.05) \| 42.12 (19.43) \| 51.05 (35.33) \| \| Bodily pain \| 63.30 (21.48) \| 72.44 (22.46) \| 72.63 (21.79) \| 78.36 (39.44) \| \| General health \| 53.26 (19.01) \| 53.98 (19.93) \| 55.83 (19.27) \| 63.24 (34.97) \| \| PCS \| 43.40 (9.02) \| 47.20 (9.47) \| 46.31 (9.12) \| 48.08 (16.67) \| \| MCS \| 45.54 (8.91) \| 43.76 (9.35) \| 40.83 (9.00) \| 49.83 (16.47) \| |
| Faulconbridge, 2012 [38] | BED | BED, bariatric surgery: 36  BED, lifestyle modification: 49 | SF-36 | \| **Baseline SF-36 scale and summary scores by study groups** \| \| \| \| --- \| --- \| --- \| \|  \| **Bariatric surgery**  mean (SE) \| **Lifestyle modification**  mean (SE) \| \| Physical functioning \| 34.9 (1.9) \| 37.3 (1.5) \| \| Physical role limitation \| 41.8 (1.7) \| 43.7 (1.5) \| \| Bodily pain \| 39.5 (1.6) \| 44.8 (1.6) \| \| General health \| 38.6 (1.7) \| 41.4 (1.4) \| \| Vitality \| 39.0 (1.6) \| 42.7 (1.4) \| \| Social functioning \| 38.3 (2.0) \| 42.7 (1.7) \| \| Emotional role limitation \| 42.3 (1.7) \| 43.7 (1.8) \| \| Mental health \| 43.5 (1.8) \| 44.8 (1.7) \| \| PCS \| 37.7 (1.7) \| 40.8 (1.3) \| \| MCS \| 43.1 (1.6) \| 45.4 (2.0) \| |
| Fox, 2009 [18] | AN | AN: 43  Control: 56 | McGill QLS | \| **McGill QLS scores by study groups** \| \| \| \| --- \| --- \| --- \| \|  \| **AN** \| **Control** \| \| McGill- existential well-being subscale \| 19.39 (9.43) \| 44.23 (9.03) \| |
| Gonzalez-Pinto, 2004 [19] | AN | AN: 47 | SF-36 | \| **SF-36 scale and summary scores** \| \| \| --- \| --- \| \| **Scales** \| **Scores** \| \| Physical functioning \| 83.98 (13.2) \| \| Role physical \| 61.70 (40.3) \| \| Bodily pain \| 72.91 (25.6) \| \| General health \| 57.42 (20.4) \| \| Vitality \| 52.34 (23.6) \| \| Social functioning \| 63.56 (29.9) \| \| Role emotional \| 65.95 (40.2) \| \| Mental health \| 49.29 (23.5) \| \| PCS \| 50.41 (8.2) \| \| MCS \| 36.25 (14.6) \| |
| Grenon, 2010 [39] | BED | BED 105 | EQ-5D-3L | \| **EQ-5D-3L index scores** \| \| \| \| --- \| --- \| --- \| \|  \| **BED** \| **US community sample of women within the mean age group of 35 to 44 years** \| \| EQ-5D index score \| 0.77 ( 0.16) \| 0.89 (0.18) \| |
| Hsu, 2002 [40] | BED | BED:9  TOTAL: 37 | SF-36 | Only descriptive results and statistical significance were presented |
| Keilen, 1994 [20] | AN, BN | AN: 62  BN: 80  Control: 95 | NHP | HRQoL results were presented only graphically. |
| Kolotkin, 2004 [41] | BED | BED: 95  Non-BED: 435 | IWQOL-Lite | \| **IWQOL-Lite scale scores by study groups** \| \| \| \| --- \| --- \| --- \| \|  \| **BED** \| **Non-BED** \| \| IWQOL total \| 51.5 (21.9) \| 65.3 (19.8) \| \| IWQOL physical function \| 49.7 (28.2) \| 59.4 (26.1) \| \| IWQOL self-esteem \| 39.3 (25.9) \| 61.6 (26.2) \| \| IWQOL sexual life \| 59.1 (30.4) \| 67.8 (27.5) \| \| IWQOL public distress \| 59.2 (28.4) \| 74.3 (25.8) \| \| IWQOL work \| 60.8 (28.9) \| 75.2 (21.0) \| |
| Latner, 2008 [21] | AN, BN, BED, EDNOS | AN: 11  BN: 5  BED: 3  EDNOS: 30 | SF-36 | No ED diagnosis-specific HRQoL results were presented |
| Marchesini, 2002 [42] | BED | BED, CBT: 92  BED, control: 76 | SF-36 | \| **SF-36 subscale and summary scores by study groups** \| \| \| \| \| \| --- \| --- \| --- \| --- \| --- \| \|  \| **CBT group** \| \| **Control group** \| \| \|  \| **Baseline** \| **End** \| **Baseline** \| **End** \| \| Physical Functioning \| 68 (23) \| 79 (21)* \| 71 (23) \| 72 (23) \| \| Role Limitation - Physical \| 60 (40) \| 79 (30)* \| 64 (40) \| 66 (39) \| \| Bodily Pain \| 60 (28) \| 69 (27)* \| 61 (27) \| 57 (26) \| \| General Health \| 55 (12) \| 60 (13)* \| 57 (10) \| 54 (9) \| \| Vitality \| 51 (21) \| 60 (19)* \| 51 (20) \| 53 (19) \| \| Social Functioning \| 63 (25) \| 71 (21)* \| 64 (26) \| 65 (23) \| \| Role Limitation - Emotional \| 59 (41) \| 76 (32)* \| 57 (41) \| 62 (40) \| \| Mental Health \| 58 (21) \| 66 (19)* \| 59 (21) \| 57 (21) \| \| PCS \| 43 (10) \| 47 (9)* \| 44 (10) \| 44 (9) \| \| MCS \| 42 (13) \| 47 (10)* \| 42 (12) \| 42 (12) \| |
| Masheb, 2004 [43] | BED | BED: 94 | SF-36 | \| **SF-36 scale scores: BED vs. US norms** \| \| \| \| --- \| --- \| --- \| \|  \| **BED** \| **US norms** \| \| Physical functioning \| 75.2 (21.5) \| 84.5 (22.8) \| \| Physical role limitation \| 67.3 (39.1) \| 81.1 (33.7) \| \| Bodily pain \| 60.7 (24.4) \| 75.4 (23.5) \| \| General health \| 65.4 (18.6) \| 72.2 (20.1) \| \| Vitality \| 39.9 (19.9) \| 61.0 (20.8) \| \| Social functioning \| 66.7 (26.5) \| 83.5 (22.3) \| \| Emotional role limitation \| 52.5 (42.2) \| 81.2 (33.0) \| \| Mental health \| 59.2 (16.6) \| 74.8 (18.0) \|  \| **SF-36 scale and summary scores: obese vs. nonobese patients with BED** \| \| \| \| --- \| --- \| --- \| \|  \| **BMI ≥30** (n=71) \| **BMI <30** (n=23) \| \| Physical functioning \| 69.9 (21.8) \| 91.5 (8.5) \| \| Physical role limitation \| 63.4 (39.8) \| 79.3 (35.1) \| \| Bodily pain \| 56.7 (23.7) \| 72.8 (22.8) \| \| General health \| 62.4 (18.8) \| 74.7 (14.6) \| \| Vitality \| 37.6 (19.5) \| 47.2 (19.9) \| \| Social functioning \| 63.3 (27.6) \| 77.2 (19.8) \| \| Emotional role limitation \| 49.8 (42.1) \| 60.9 (42.2) \| \| Mental health \| 58.2 (15.9) \| 62.3 (18.6) \| \| PCS \| 45.3 (9.6) \| 53.6 (9.4) \| \| MCS \| 39.3 (10.6) \| 41.0 (12.2) \| |
| Mond, 2005 [22] | AN, BN, BED | AN-P: 15  AN-R: 19  BN: 40  BED: 10 | SF-12, WHOQOL-BREF | \| **SF-12 summary scores by diagnostic groups** \| \| \| \| \| \| --- \| --- \| --- \| --- \| --- \| \|  \| **AN-P** \| **AN-R** \| **BED** \| **BN** \| \| MCS \| 26.96 (7.44) \| 38.38 (11.05) \| 30.36 (7.96) \| 27.60 (9.44) \| \| PCS \| 46.80 (10.03) \| 45.38 (10.28) \| 40.18 (13.11) \| 49.25 (10.07) \|  \| **WHOQOL-BREF scale scores by diagnostic groups** \| \| \| \| \| \| --- \| --- \| --- \| --- \| --- \| \|  \| **AN-P** \| **AN-R** \| **BED** \| **BN** \| \| QoLP \| 2.07 (0.73) \| 2.71 (0.91) \| 2.17 (0.47) \| 2.42 (0.71) \| \| QoLS \| 2.58 (1.06) \| 3.58 (1.01) \| 2.20 (0.98) \| 3.09 (0.96) \| |
| Mond, 2010 [32] | patients with objective or subjective bulimic episodes | Objective bulimic episodes: 37  Subjective bulimic episodes: 52  Objective and subjective bulimic episodes: 13 | SF-12 | \| **SF-12 summary scores by study groups** \| \| \|  \| \| --- \| --- \| --- \| --- \| \|  \| **Objective bulimic episodes** \| **Subjective bulimic episodes** \| **Objective and subjective bulimic episodes** \| \| PCS \| 46.50 (10.21) \| 48.84 (10.82) \| 45.77 (11.64) \| \| MCS \| 36.34 (11.98) \| 36.28 (12.23) \| 36.58 (10.86) \| |
| Munoz, 2009 [23] | AN, BN, EDNOS | AN: 61  BN: 47  EDNOS: 245 | SF-12,  HeRQoLED | SF-12: No ED diagnosis-specific scores were presented   \| **HeRQoLED domain scores by initial diagnostic category** \| \| \| \| \| \| \| \| --- \| --- \| --- \| --- \| --- \| --- \| --- \| \|  \| **AN** \| \| **BN** \| \| **EDNOS** \| \| \|  \| **At baseline** \| **After 1 year of treatment** \| **At baseline** \| **After 1 year of treatment** \| **At baseline** \| **After 1 year of treatment** \| \| Symptoms \| 42.23 (18.69) \| 37.73 (18.06) \| 52.75 (17.65) \| 40.75 (18.50) \| 35.24 (19.11) \| 30.92 (19.42) \| \| Restrictive behavior \| 35.16 (25.33) \| 28.06 (25.71) \| 38.88 (17.65) \| 37.48 (26.62) \| 26.21 (23.12) \| 22.01 (24.79) \| \| Body image \| 60.05 (25.63) \| 59.80 (25.63) \| 71.90 (25.64) \| 67.90 (27.29) \| 54.71 (25.79) \| 50.89 (25.79) \| \| Mental health \| 55.42 (21.35) \| 54.98 (24.07) \| 58.36 (17.20) \| 51.28 (18.42) \| 48.46 (19.67) \| 45.93 (20.19) \| \| Emotional role \| 42.23 (27.91) \| 41.81 (27.53) \| 49.53 (23.26) \| 38.91 (25.10) \| 38.58 (27.68) \| 32.85 (25.19) \| \| Physical role \| 29.50 (25.58) \| 33.75 (27.90) \| 36.77 (29.26) \| 32.26 (20.69) \| 28.05 (25.83) \| 24.76 (23.89) \| \| Personality traits \| 58.33 (26.86) \| 57.71 (27.99) \| 63.84 (20.00) \| 56.50 (21.11) \| 50.80 (24.19) \| 46.43 (22.83) \| \| Social relations \| 54.23 (29.13) \| 49.38 (24.67) \| 61.28 (26.87) \| 53.39 (31.16) \| 39.18 (28.40) \| 32.34 (27.41) \| |
| Nickel, 2005 [33] | BN | BN, topiramate group: 30  BN, placebo group: 30 | SF-36 | \| **SF-36 scale scores by study groups** \| \| \| \| \| \| --- \| --- \| --- \| --- \| --- \| \|  \| **Topiramate group** \| \| **Placebo group** \| \| \|  \| **Baseline** \| **Final** \| **Baseline** \| **Final** \| \| Physical functioning \| 67.2 (6.5) \| 71.2 (6.6) \| 65.4 (5.8) \| 66.0 (5.6) \| \| Physical role limitation \| 57.1 (6.0) \| 60.5 (5.9) \| 57.1 (5.3) \| 57.6 (5.5 \| \| Bodily pain \| 59.8 (7.2) \| 62.0 (7.6) \| 61.5 (7.0) \| 61.8 (7.2) \| \| General health \| 51.0 (5.3) \| 60.7 (5.6) \| 53.6 (5.5) \| 53.7 (5.7) \| \| Vitality \| 52.3 (4.1) \| 60.5 (6.0) \| 53.3 (5.3) \| 53.5 (5.5) \| \| Social functioning \| 64.9 (6.2) \| 69.6 (9.5) \| 64.1 (7.7) \| 64.1 (7.6) \| \| Emotional role limitation \| 61.2 (8.0) \| 68.1 (7.4) \| 57.6 (8.2) \| 57.9 (7.9) \| \| Mental health \| 60.9 (6.1) \| 66.2 (6.5) \| 60.4 (6.2) \| 60.5 (6.2) \| |
| Padierna, 2000 [24] | AN, BN, BED | AN-R: 56  AN-P: 60  BN: 64  BED: 17 | SF-36 | \| **SF-36 scale scores by diagnostic groups** \| \| \| \| \| \| --- \| --- \| --- \| --- \| --- \| \|  \| **AN-R** \| **AN-P** \| **BN** \| **BED** \| \| Physical functioning \| 87.5 (15.4) \| 88.9 (16.4) \| 89.5 (11.9) \| 72.3 (22.7) \| \| Physical role limitation \| 62.0 (40.6) \| 52.1 (43.3) \| 54.8 (41.4) \| 46.9 (42.7) \| \| Bodily pain \| 72.4 (22.7) \| 64.9 (30.1) \| 63.3 (28.0) \| 60.7 (30.9) \| \| General health \| 51.6 (19.1) \| 50.1 (23.1) \| 47.1 (21.0) \| 50.7 (23.1) \| \| Vitality \| 51.7 (22.0) \| 44.5 (23.5) \| 44.9 (20.8) \| 40.9 (21.5) \| \| Social functioning \| 62.3 (30.9) \| 51.5 (30.1) \| 54.5 (28.1) \| 52.2 (25.9) \| \| Emotional role limitation \| 57.6 (42.3) \| 41.7 (42.8) \| 41.8 (43.6) \| 37.3 (45.5) \| \| Mental health \| 47.8 (23.2) \| 41.5 (23.4) \| 46.4 (23) \| 44.5 (16.0) \| |
| Perez, 2012 [44] | BED | BED, obese+nonobese: 250 | self-developed instrument | The HRQoL results cannot be generalized |
| Pohjolainen, 2010 [34] | BN | BN: 72 | 15D | \| **15D scores** \| \| \| \| --- \| --- \| --- \| \|  \| **Treated BN patients** \| \| \|  \| **Baseline** \| **After 6 months** \| \| HRQoL scores \| 0.80 (0.09) \| 0.85 (0.10) \| |
| Ricca, 2009 [45] | BED | BED: 105  BED, subthreshold: 146  Overweight non-BED: 187 | ORWELL-97 | \| **ORWELL-97 total scores** \| \| \| \| \| --- \| --- \| --- \| --- \| \|  \| **BED** \| **BED subthreshold** \| **Overweight non-BED** \| \| ORWELL-97 total \| 54.3 (21.2) \| 53.0 (20.3) \| 56.0 (22.2) \| |
| Rie, 2005 [25] | AN  BN  EDNOS | AN: 44  BN: 43  EDNOS: 69 | SF-36 | \| **SF-36 scale scores by diagnostic groups** \| \| \| \| \| --- \| --- \| --- \| --- \| \|  \| **AN** \| **BN** \| **EDNOS** \| \| Physical functioning \| 80.2 (18.2) \| 84.5 (15.4) \| 81.7 (22.1) \| \| Physical role functioning \| 42.0 (37.7) \| 44.6 (39.2) \| 47.7 (43.6) \| \| Bodily pain \| 65.9 (23.1) \| 67.8 (19.6) \| 64.6 (26.3) \| \| General health perception \| 48.8 (21.3) \| 52.5 (17.5) \| 52.3 (21.5) \| \| Vitality \| 39.5 (17.7) \| 36.2 (15.7) \| 41.4 (16.1) \| \| Social functioning \| 46.6 (22.5) \| 42.2 (26.4) \| 52.4 (23.7) \| \| Emotional role functioning \| 29.5 (36.8) \| 22.2 (32.6) \| 27.8 (37.2) \| \| Mental health \| 41.6 (16.6) \| 38.3 (16.9) \| 44.1 (16.3) \| |
| Rieger, 2005 [46] | BED | BED: 56  Non-BED: 62 | IWQOL-LITE | \| **IWQOL-LITE scale scores by study groups** \| \| \| \| --- \| --- \| --- \| \|  \| **BED** \| **Non-BED** \| \| Work \| 7.75 (3.16) \| 6.53 (2.75) \| \| Public Distress \| 10.13 (4.08) \| 8.48 (3.72) \| \| Sexual Life \| 9.14 (3.57) \| 7.61 (3.51) \| \| Physical Function \| 25.23 (10.02) \| 20.98 (16.27) \| \| Self-Esteem \| 21.79 (6.74) \| 17.53 (6.65) \| \| Total \| 74.04 (19.29) \| 61.15 (26.31) \| |
| Silveira, 2005 [47] | BED | BED: 9 | WHOQOL-BREF | No detailed HRQoL results were presented |
| Thien, 2000 [26] | AN | AN, exercise group: 5  AN, control group: 7 | SF-36 | \| **Baseline SF-36 scale scores by study groups** \| \| \| \| --- \| --- \| --- \| \|  \| **Exercise group** \| **Control group** \| \| SF-36 summary \| 58.8 (13.9) \| 53.3 (14.5) \| \| Role Physical component score \| 55.0 (37.1) \| 50.0 (47.9) \| \| Social Functioning component score \| 72.5 (18.5) \| 62.5 (14.4) \| \| Vitality component score \| 37.0 (28.2) \| 39.3 (24.4) \| \| Sum of three scales \| 54.8 (20.1) \| 50.6 (22.5) \|  \| **Changes in SF-36 scale scores by study groups** \| \| \| \| --- \| --- \| --- \| \|  \| **Exercise group** \| **Control group** \| \| ΔSF-36 \| 6.6 (7.0) \| −12.0 (25.5) \| \| Δ Role Physical \| 25.0 (35.4) \| −10.7 (53.7) \| \| Δ Social Functioning \| 5.0 (18.9) \| −19.6 (27.8) \| \| Δ Vitality \| 5.0 (25.7) \| −2.8 (32.3) \| \| ΔΣ3-scales \| 11.7 (19.5) \| −11.0 (34.2) \| |
| Turner, 2010 [27] | AN  BN  EDNOS | AN: 14  BN: 66  EDNOS: 98 | SF-36 | \| **SF-36 scale scores by diagnostic groups** \| \| \| \| \| --- \| --- \| --- \| --- \| \|  \| **AN** \| **BN** \| **EDNOS** \| \| Physical functioning \| 68.9 (21.5) \| 80.0 (25.4) \| 83.2 (21.7) \| \| Physical role limitation \| 14.3 (25.4) \| 57.1 (44.0) \| 70.0 (38.5) \| \| Bodily pain \| 42.0 (22.0) \| 60.8 ( 24.7) \| 68.0 (25.5) \| \| General health \| 35 (18.8) \| 42.9 (22.0) \| 48.7 (24.0) \| \| Vitality \| 18.2 (14.9) \| 27.4 (17.1) \| 33.2 (21.2) \| \| Social functioning \| 32.5 (24.8) \| 49.1 (30.6) \| 52.6 (26.9) \| \| Emotional role limitation \| 21.4 (28.0) \| 30.8 (37.9) \| 28.6 (36.8) \| \| Mental health \| 30.9 (22.0) \| 34.2 (17.8) \| 40.0 (19.1) \| |
| Walsh, 2006 [28] | AN | AN, fluoxetine group: 49  AN, placebo group: 44 | QLES-Q | \| **QLES-Q scores by study groups** \| \| \| \| \| \| --- \| --- \| --- \| --- \| --- \| \|  \| **Baseline** \| \| **At termination** \| \| \|  \| **Fluoxetine** \| **Placebo** \| **Fluoxetine** \| **Placebo** \| \| QLES-Q \| 49.63 (11.16) \| 52.07 (8.82) \| 51.70 (12.33) \| 52.15 (13.62) \| |
| Watson, 2012 [29] | AN, BN, EDNOS, | AN: 34  BN: 87  EDNOS: 75 | Q-LES-Q SF | \| **Q-LES-Q SF scores by diagnostic groups** \| \| \| \| \| \| \| \| --- \| --- \| --- \| --- \| --- \| --- \| --- \| \|  \| **AN** \| \| **BN** \| \| **EDNOS** \| \| \|  \| **Pre-treatment** \| **Post-treatment** \| **Pre-treatment** \| **Post-treatment** \| **Pre-treatment** \| **Post-treatment** \| \| Q-LES-Q SF \| 51.85 (22.28) \| 66.54 (19.02) \| 49.43 (16.05) \| 64.96 (15.39) \| 50.23 (14.07) \| 68.17 (16.24) \| |
| Watson, 2013 [30] | AN, BN | AN-R: 45  AN-P: 24  Objective BN: 112  Subjective BN: 28 | Q-LES-Q SF | \| **Q-LES-Q SF score by diagnostic groups** \| \| \| \| \| \| --- \| --- \| --- \| --- \| --- \| \|  \| **AN-R** \| **AN-P** \| **Objective BN** \| **Subjective BN** \| \| Q-LES-Q SF \| 52.05 (18.4) \| 38.22 (14.58) \| 46.59 (15.57) \| 46.29 (21.13) \| |
| Wilfley, 2008 [48] | BED | BED, sibutramine group: 152  BED, placebo group: 152 | IWQOL-Lite | \| **IWQOL-Lite total scores by study groups** \| \| \| \| \| \| --- \| --- \| --- \| --- \| --- \| \|  \| **Baseline** \| \| **After 24 weeks (completers)** \| \| \|  \| **Sibutramine** \| **Placebo** \| **Sibutramine** \| **Placebo** \| \| IWQOL-Lite \| 67.7 (18.2) \| 68.7 (18.5) \| 77.2 (15.4) \| 72.2 (17.1) \| |

AN: anorexia nervosa, AN-R: anorexia nervosa restricting type, AN-P: anorexia nervosa purging type, BED: binge eating disorder, BN: bulimia nervosa, CBT: cognitive behavioral therapy, ED: eating disorder, EDNOS: eating disorder not otherwise specified, EDQoL: Eating Disorders Quality of Life, EQ-5D-3L: EuroQol Five Dimensional Questionnaire, ESWLS: Extended Satisfaction With Life Scale, HeRQoLED: Health-related Quality of Life for Eating Disorders, HRQoL: health-related quality of life, IWQOL-LITE: Impact of Weight on Quality of Life – Lite, McGill-QLS: McGill Quality of Life Scale, MCS: Mental Component Summary, NHP: Nottingham Health Profile, ORWELL-97: Obesity Related Well-Being 97, PCS: Physical Component Summary, QLES-Q: Quality of Life Enjoyment and Satisfaction Questionnaire, QLES-Q SF: QLES-Q Short Form, QOL-ED: Quality of Life Eating Disorder, QoLP: WHOQOL-BREF Psychological Health Scale, QoLS: WHOQOL-BREF Social Relationships Scale, SF-12: Medical Outcomes Study Short-Form 12, SF-36: Medical Outcomes Study Short-Form 36, WHOQOL-BREF: World Health Organization Brief Quality of Life Assessment Scale, 15-D: 15-Dimensional
