# Supplementary material for: A systematic review of the health-related quality of life and economic burdens of anorexia nervosa, bulimia nervosa, and binge eating disorder
Source: Eat Weight Disord. 2016 Mar 4;21(3):353–64. doi: 10.1007/s40519-016-0264-x (PMC5010619; doi:10.1007/s40519-016-0264-x)
Supplement: Supplementary file 4 — Supplementary material 4 (DOCX 53 kb) [file 40519_2016_264_MOESM4_ESM.docx]

# **Online Resource 4.** Selected healthcare utilization data for AN, BN, and BED

| **First author, year** | **Included eating disorders: AN, BN and BED** | **Reported healthcare utilization data** |
| --- | --- | --- |
| Calderon, 2007 [54] | AN, BN | \| *Inpatient care* \|  \|  \| \| --- \| --- \| --- \| \|  \| **AN** \| **BN** \| \| Hospital unit % \|  \|  \| \| Psychiatric  Intensive care  Medical unit  Other/unknown \| 51%  8%  13%  32% \| 68%  2%  2%  25% \| \| Length of hospitalization mean(SD) \| 17.5(18.9) \| 12.9(9.8) \| \| Inpatient medications % \|  \|  \| \| Any psychotropic  Antidepressant  SSRI  Anti-anxiety  Atypical or typical Anti-psychotic \| 69%  59%  55%  19%  23% \| 78%  70%  67%  26%  11% \| |
| Dickerson, 2011 [68] | BED | \| *% with any use* \| \| \| \| --- \| --- \| --- \| \|  \| **BED** \| **Recurrent binge eating** \| \| Weight- and eating disorder-related services \| 24% \| 18% \| \| Non–weight- and eating disorder-related mental health services \| 24% \| 22% \| \| Other provider-based services \| 100% \| 98% \| \| Mental health medication services \| 62% \| 54% \| \| Total medication services \| 90% \| 90% \| \| Total health services \| 100% \| 98% \| |
| George, 1987 [55] | AN | \|  \| **1958-1962** \| **1968-1972** \| **1978-1982** \| \| --- \| --- \| --- \| --- \| \| Length of hospitalization mean \| 24 days \| 26 days \| 131 days \| \| Treatment % \|  \|  \|  \| \| Psychotherapy  Behavior  Group  Family  Antidepressant  Antipsychotic \| 52%  0%  8%  4%  4%  0% \| 68%  4%  9%  23%  24%  28% \| 100%  84%  48%  71%  58  19% \| |
| Grenon, 2010 [39] | BED | \| ***% of participants endorsing each healthcare domain*** \| \| \| --- \| --- \| \| Family physician visits  Medication use  Diagnostic tests  Health professionals’ visits  Specialist visits  Herbal remedies  Other resources  Outpatient visits  Emergency department visits  Inpatient visits \| 82%  72%  53%  51%  49%  34%  16%  15%  13%  2% \| |
| Grigoriadis, 2001 [56] | AN | \| ***Healthcare utilization of patients within the first 6 months following discharge*** \| \| \| --- \| --- \| \| Family doctor  Psychiatrist  Family doctor sessions  Psychiatrist sessions  Medication  Total psychotropic  Antidepressant  Antianxiety  Antipsychotic \| 75%  46%  89 sessions/patient  19 sessions/patient  88%  ~80%  ~40%  ~10% \| |
| Jager, 2004 [67] | BN | \|  \| **Systematic outpatient therapy** \| **Analytic inpatient therapy** \| \| --- \| --- \| --- \| \| Inpatient treatments days  0  <30  >30 \| 65%  13%  22% \| 65%  0%  35% \| \| Outpatient treatments month  0  <6  >6  >12 \| 37%  15%  11%  37% \| 35%  3%  5%  57% \| \| Psychotherapeutic sessions n  0  <20  <120  >120 \| 37%  20%  33%  11% \| 35%  0%  35%  30% \| |
| Kalisvaart, 2007 [57] | AN | \| Length of hospital stay per case mean \| 51 days \| \| --- \| --- \| |
| Kessler, 2013 [2] | BN, BED | \|  \| **BN** \| **BED** \| \| --- \| --- \| --- \| \| Lifetime treatment for emotional problems \| 67% \| 58% \| \| 12-month treatment for emotional problems \| 45% \| 37% \| \| Lifetime treatment for eating disorders \| 47% \| 38% \| \| 12-month treatment for eating disorders \| 26% \| 10% \| |
| Krauth, 2002 [58] | AN, BN | \|  \| **AN** \| **BN** \| \| --- \| --- \| --- \| \| Inpatient treatment days/case \| 49.8 \| 45.5 \| \| Inability to work days/case \| 78.4 \| 28.8 \| |
| Marques, 2011 [65] | AN, BN, BED | \| ***Lifetime any service use*** \| \| \| \| \| --- \| --- \| --- \| --- \| \|  \| AN \| BN \| BED \| \| Non-Latino White \| 76% \| 77% \| 79% \| \| Latino \| 79% \| 43% \| 54% \| \| Asian \| 30% \| 39% \| 55% \| \| African American \| 100% \| 80% \| 71% \| |
| Mond, 2007 [59] | AN, BN, BED | \| ***Lifetime service use*** \| \| \| \| \| --- \| --- \| --- \| --- \| \|  \| **AN** \| **BN** \| **BED** \| \| Any treatment  Eating  General mental health  Weight \| 61%  78%  50% \| 37%  71%  71% \| 58%  84%  87% \| \| Treatment by a mental health professional  Eating  General mental health  Weight \| 22%  22%  39% \| 14%  33%  35% \| 23%  39%  45% \| |
| O’Brien, 2003 [60] | AN, BN | \|  \| **AN** \| **BN** \| \| --- \| --- \| --- \| \| Length of hospital stay mean \| 15 days \| 9 days \| |
| Pohjolainen, 2010 [34] | BN | \| Length of inpatient treatment during 6 months \| 8 days \| \| --- \| --- \| \| Number of outpatients visits during 6 moths \| 15 visits \| |
| Preti, 2009 [3] | AN, BN, BED | \|  \| **AN** \| **BN** \| **BED** \| \| --- \| --- \| --- \| --- \| \| *Lifetime access to service use for any emotional problem* \| \| \| \| \| General medical \| 35% \| 49% \| 24% \| \| Psychiatrist \| 14% \| 32% \| 16% \| \| Other mental health \| 22% \| 40% \| 18% \| \| Non-medical professional \| 0% \| 4% \| 6% \| \| Complementary-alternative medical \| 8% \| 2% \| 12% \| \| Any lifetime treatment \| 52% \| 55% \| 39% \| \| *12-month access to service use for any emotional problem* \| \| \| \| \| General medical \| n.a. \| 59% \| 17% \| \| Psychiatrist \| n.a. \| 12% \| 10% \| \| Other mental health \| n.a. \| 3% \| 3% \| \| Non-medical professional \| n.a. \| 0% \| 10% \| \| Complementary-alternative medical \| n.a. \| 3% \| 1% \| \| Any 12-month treatment \| n.a. \| 60% \| 23% \| |
| Striegel-Moore, 2000 [61] | AN, BN | \|  \| **AN** \| **BN** \| \| --- \| --- \| --- \| \| *Inpatient treatment days per year mean(SD)* \| \| \| \| Female \| 26.0(29.7) \| 14.7(13.8) \| \| Male \| 15.6(18.3) \| 21.7(21.8) \| \| *Outpatient treatment days per year mean(SD)* \| \| \| \| Female \| 17.0(22.4) \| 15.6(18.1) \| \| Male \| 9.2(11.2) \| 9.1(9.6) \| |
| Striegel-Moore, 2004 [69] | BED | \| ***12-month data*** \| **BED** \| \| \| \| **Healthy comparison** \| \| \| \| **Psychiatric comparison** \| \| \| \| \| --- \| --- \| --- \| --- \| --- \| --- \| --- \| --- \| --- \| --- \| --- \| --- \| --- \| \|  \| **White** \| \| **Black** \| \| **White** \| \| **Black** \| \| **White** \| \| **Black** \| \| \|  \| Obese \| Non-  obese \| Obese \| Non-  obese \| Obese \| Non-  obese \| Obese \| Non-  obese \| Obese \| Non-  obese \| Obese \| Non-  obese \| \| Outpatient psychotherapist visits % \| 33% \| 24% \| 24% \| 27% \| 0% \| 3% \| 0% \| 7% \| 54% \| 56% \| 25% \| 23% \| \| Emergency department visits % \| 40% \| 24% \| 16% \| 33% \| 17% \| 13% \| 14% \| 14% \| 23% \| 21% \| 38% \| 62% \| \| Inpatient days % \| 11% \| 11% \| 7% \| 13% \| 11% \| 8% \| 14% \| 4% \| 31% \| 10% \| 25% \| 23% \| \| Outpatient physician visits mean(SD) \| 7.9 (10.5) \| 4.1  (5.8) \| 4.4 (8.2) \| 7.1  (10.1) \| 6.2 (8.6) \| 3.1 (3.6) \| 4.1 (7.7) \| 3.7  (5.3) \| 8.2 (10.8) \| 6.0  (8.1) \| 3.3 (3.0) \| 4.4  (4.4) \| \| Total service days mean(SD) \| 21.4 (28.1) \| 11.8 (21.8) \| 17.6 (30.1) \| 14.9 (23.0) \| 8.4 (12.7) \| 3.4 (3.9) \| 4.6 (8.4) \| 4.8  (7.9) \| 16.1 (14.0) \| 18.4  (23.7) \| 6.9 (4.6) \| 8.5 (10.8) \| |
| Striegel-Moore, 2008 [62] | AN, BN | \| *12-month data* \| \| \| \|  \| \| \| \| --- \| --- \| --- \| --- \| --- \| --- \| --- \| \|  \| **AN** \| \| \| **BN** \| \| \| \| Age group \| 18-24 \| 25-34 \| 35-55 \| 18-24 \| 25-34 \| 35-55 \| \| Phone consultations mean(SD) \| 5.1(10.4) \| 1.9(5.3) \| 21.3(34.5) \| 4.0(6.0) \| 7.7(15.1) \| 7.8(10.1) \| \| Mental health mean(SD) \| 2.4(4.4) \| 1.7(2.9) \| 17.0(25.1) \| 2.3(3.4) \| 4.1(5.8) \| 7.1(11.3) \| \| Anxiolytics mean(SD) \| -0.1(0.13) \| -0.1(0.1) \| 0.7(0.5) \| -0.1(0.2) \| 0.2(0.5) \| 0.3(0.5) \| |
| Swanson, 2011 [66] | AN, BN, BED | \| ***Lifetime service use*** \| \| \| \| \| \| --- \| --- \| --- \| --- \| --- \| \|  \| **AN** \| **BN** \| **BED** \| **No-eating disorder** \| \| Mental health specialty \| 69% \| 61% \| 60% \| 29% \| \| General medicine \| 27% \| 49% \| 22% \| 12% \| \| Human service \| 40% \| 30% \| 18% \| 10% \| \| Complementary and alternative medicine \| 24% \| 14% \| 4% \| 8% \| \| Juvenile justice \| 11% \| 2% \| 1% \| 5% \| \| School service \| 40% \| 45% \| 20% \| 21% \| \| Any treatment \| 78% \| 88% \| 73% \| 44% \| \| Treatment specifically for eating or weight problems \| 28% \| 22% \| 11% \| n.a. \| |
| Wales, 2013 [63] | AN | \| ***10-year retrospective case note study*** \|  \|  \| \| --- \| --- \| --- \| \|  \| **AN restrictive** \| **AN binge/purge** \| \| Number of total sessions mean(range) \| 53.3(6-283) \| 32.0(1-123) \| \| Number of months in contact mean(range) \| 56.6(6-192) \| 33.4(1-129) \| |
| Williamson, 2001 [64] | AN, BN | \|  \| **AN** \| **BN** \| \| --- \| --- \| --- \| \| Length of hospital stay in days mean(SD) \| 52.7(39.5) \| 45.7(34.9) \| |

AN: anorexia nervosa, BED: binge eating disorder, BN: bulimia nervosa, n.a.: not applicable
