# Supplementary material for: A systematic review of the health-related quality of life and economic burdens of anorexia nervosa, bulimia nervosa, and binge eating disorder
Source: Eat Weight Disord. 2016 Mar 4;21(3):353–64. doi: 10.1007/s40519-016-0264-x (PMC5010619; doi:10.1007/s40519-016-0264-x)
Supplement: Supplementary file 5 — Supplementary material 5 (DOCX 55 kb) [file 40519_2016_264_MOESM5_ESM.docx]

# **Online Resource 5.** Selected healthcare cost data for AN, BN and BED

| **First author, year** | **Perspective** | **Country, year of pricing** | **Cost categories and reported costs** | | **Annual costs per patient in 2014 EUR** |
| --- | --- | --- | --- | --- | --- |
|  |  |  |  |  |  |
| **AN** | | | | | |
| Byford, 2007 [70] | payer | UK  2004 | \|  \| Inpatient mean(SD) \| Specialist outpatient mean(SD) \| General outpatient mean(SD) \| \| --- \| --- \| --- \| --- \| \| Secondary healthcare^a^ \| £32,015(51,541) \| £24,724(46,231) \| £37,746(62,046) \| \| Primary healthcare \| £380(640) \| £385(873) \| £245(361) \| \| Education^b^ \| £2,098(2,115) \| £1,595(1,456) \| £2,654(2,228) \| \| Other community services^c^ \| £37(110) \| £35(104) \| £150(806) \| \| Total 2-year costs \| £34,531(52,439) \| £26,738(46,809) \| £40,794(63,652) \|   a: inpatient nights, outpatient appointments, day patient contacts, accident an emergency contacts  b: state day school, independent day school, independent boarding school, hospital school, home tuition, school counselor, education welfare officer  c: general practitioner, practice nurse, dietician, district nurse, health visitor, community pediatrician, community psychiatric nurse, clinical psychologist, counselor, family therapist, dentist, school doctor, school nurse, social worker, eating disorders association, family therapy, foster care | | \| Inpatient care \| €23,607 \| \| --- \| --- \| \| Specialist outpatient care \| €18,280 \| \| General outpatient care \| €27,889 \| |
| Crow, 2004 [71] | payer | US  2004 | \|  \| Usual care \| Adequate care \| \| --- \| --- \| --- \| \| Inpatient treatment \| $14,000 \| $90,000 \| \| Partial hospitalization \| $12,000 \| $16,000 \| \| Psychotherapy \| $3,000 \| $6,000 \| \| Medication management \| $1,800 \| $1,800 \| \| Fluoxetine (60mg/days) \| $5,400 \| $5,400 \| \| Total 2-year costs \| $36,200 \| $119,200 \| | | \| Usual care \| €16,785 \| \| --- \| --- \| \| Adequate care \| €55,270 \| |
| Haas, 2012 [72] | hospital | Germany  2007 | \| Inpatient cost^a^ mean(SD) \| €4,647(3,714) \| \| --- \| --- \|   a: physicians, nursing, psychotherapists, dietician, art and music therapy, psychotherapy, drugs, medical products, services on demand, overhead costs (medical/non-medical infrastructure) | | €4,900 |
| Haas, 2012 [73] | hospital | Germany  2008 | \| Inpatient cost^a^ \| €5,251 \| \| --- \| --- \|   a: physicians, nursing, psychotherapists, dietician, art and music therapy, psychotherapy, drugs, medical products, services on demand, overhead costs (medical/non-medical infrastructure) | | €5,445 |
| Krauth, 2002 [58] | societal | Germany  1998 | \| Cost of illness \| €5,300 \| \| --- \| --- \|   a: inpatient treatment, convalescence statutory health insurance, rehabilitation statutory pension insurance, inability to work, death | | €5,952 |
| Lock, 2008 [74] | payer | US  2004 | \| Baseline medical hospital \| 28% of total costs \| \| --- \| --- \| \| Within treatment medical hospital \| 44% of total costs \| \| Outpatient family therapy \| 16% of total costs \| \| Medication visits \| 2% of total costs \| \| Medications \| 1% of total costs \| \| Doctor visit \| 95% of total costs \| \| Total cost \| $33,105 \| | | €30,700 |
| Mitchell, 2009 [75] | payer | US  2003 | \| Hospital care \| $1,193 \| \| --- \| --- \| \| Healthcare provider \| $1,720 \| \| Prescription medication \| $492 \| \| Total \| $3,405 \| | | €3,221 |
| O'Brien, 2003 [60] | payer | US  2002 | \| Inpatient cost \| $19,354 \| \| --- \| --- \| | | €18,587 |
| Striegel-Moore, 2000 [61] | payer | US  1995 | \|  \| Female \| Male \| \| --- \| --- \| --- \| \| Total costs^a^ mean(SD) \| $6,045(15,071) \| $2,746(5,476) \|   a: inpatient treatment, outpatient treatment | | \| Female \| €6,590 \| \| --- \| --- \| \| Male \| €2,993 \| |
| **BN** | | | | | |
| Crow, 2009 [77] | societal | US  2005 | \|  \| Face-to-face cognitive behavioral therapy \| Telemedicine cognitive behavioral therapy \| \| --- \| --- \| --- \| \| Evaluation and laboratory \| $152 \| $152 \| \| Cognitive behavioral therapy \| $1,131 \| $1,342 \| \| Subject travel \| $174 \| $102 \| \| Gasoline \| $89 \| $52 \| \| Therapist travel \| $1,138 \| $0 \| \| Total costs \| $2,684 \| $1,648 \| | \| Face-to-face cognitive behavioral therapy \| €2,423 \| \| --- \| --- \| \| Telemedicine cognitive behavioral therapy \| €1,488 \| | |
| Crow, 2009 [76] | patient | US  2007 | \| Total food cost mean(SD) \| $5,582(2,812) \| \| --- \| --- \| \| Binge/purge food cost mean(SD) \| $1,599(1,155) \| \| Objective binge eating food cost mean(SD) \| $788(876) \| \| Diet/laxative/diet pill cost mean(SD) \| $237(487) \| | \| Total food cost \| €4,735 \| \| --- \| --- \| \| Binge/purge food cost \| €1,357 \| \| Objective binge eating food cost \| €668 \| \| Diet/laxative/diet pill cost \| €201 \| | |
| Crow, 2013 [31] | payer | US  2005 | \|  \| Cognitive behavioral therapy \| Stepped care \| \| --- \| --- \| --- \| \| Cognitive behavioral therapy \| $1,328 \| $509 \| \| Self-help \| $0 \| $415 \| \| Medication \| $1,112 \| $904 \| \| Physician visits \| $24 \| $49 \| \| Emergency room \| $465 \| $572 \| \| Hospitalization \| $288 \| $309 \| \| Individual therapy \| $160 \| $217 \| \| Group therapy \| $25 \| $41 \| \| Medication management \| $248 \| $113 \| \| Total costs \| $3,650 \| $3,129 \| | \| Cognitive behavioral therapy \| €3,294 \| \| --- \| --- \| \| Stepped care \| €2,824 \| | |
| Koran, 1995 [78] | payer | US  1993 | \| Cognitive behavioral therapy^a^ \| $1,545 \| \| --- \| --- \| \| Med16^a^ \| $779 \| \| Med24^a^ \| $991 \| \| Combi16^a^ \| $2,204 \| \| Combi24^a^ \| $2,416 \|   a: cost was calculated by multiplying the clinic’s professional fees by the number of visits and adding cost of the patient’s expected medication and serum despiramine level | \| Cognitive behavioral therapy \| €1,761 \| \| --- \| --- \| \| Med16 \| €888 \| \| Med24 \| €1,130 \| \| Combi16 \| €2,512 \| \| Combi24 \| €2,754 \| | |
| Krauth, 2002 [58] | societal | Germany  1998 | \| Cost of illness per year \| €1,300 \| \| --- \| --- \|   a: inpatient treatment, convalescence statutory health insurance, rehabilitation statutory pension insurance, inability to work, death | €1,460 | |
| Haas, 2012 [73] | hospital | Germany  2008 | \| Inpatient cost^a^ \| €3,265 \| \| --- \| --- \|   a: physicians, nursing, psychotherapists, dietician, art and music therapy, psychotherapy, drugs, medical products, services on demand, overhead costs (medical/non-medical infrastructure) | €3,386 | |
| Mitchell, 2009 [75] | payer | US  2003 | \| Hospital care \| $2,962 \| \| --- \| --- \| \| Healthcare provider \| $2,660 \| \| Prescription medication \| $1,017 \| \| Total \| $6,639 \| | €6,279 | |
| O'Brien, 2003 [60] | payer | US  2002 | \| Inpatient cost \| $11,877 \| \| --- \| --- \| | €11,406 | |
| Pohjolainen, 2010 [34] | payer | Finland  2002 | \| Inpatient cost (6-month) mean(SD) \| €1,986(5,006) \| \| --- \| --- \| \| Outpatient cost (6-month) mean(SD) \| €1,879(1,563) \| \| Laboratory cost (6-month) mean(SD) \| €84(100) \| \| Radiology cost (6-month) mean(SD) \| €19(64) \| \| Total costs (6-month) mean(SD) \| €3,972(5,518) \| | €7,756 | |
| Striegel-Moore, 2000 [61] | payer | US  1995 | \|  \| Female \| Male \| \| --- \| --- \| --- \| \| Total costs^a^ mean(SD) \| $2,962(5,963) \| $3,885(7,138) \|   a: inpatient treatment, outpatient treatment | \| Female \| €3,229 \| \| --- \| --- \| \| Male \| €4,235 \| | |
| Wang, 2011 [79] | payer | US  2010 | \| 2-year intervention costs of Planet Health \| $46,803 \| \| --- \| --- \|   a: school based obesity prevention program | €18,823 | |
| **BED** | | | | | |
| Dickerson, 2011 [68] | payer | US  2006 | \|  \| BED \| Recurrent binge eating \| \| --- \| --- \| --- \| \| Weight- and eating disorder-related services mean(SD) \| $72(176) \| $94(299) \| \| Non–weight- and eating disorder-related mental health services mean(SD) \| $415(1,014) \| $341(840) \| \| Other provider-based services mean(SD) \| $1,925(2,761) \| $2,221(3,818) \| \| Mental health medication services mean(SD) \| $411(695) \| $301(637) \| \| Total medication services mean(SD) \| $906(1,475) \| $933(2,087) \| \| Total costs mean(SD) \| $3,319(4,050) \| $3,588(4,665) \| | \| BED \| €2,902 \| \| --- \| --- \| \| Recurrent binge eating \| €3,137 \| | |
| Grenon, 2010 [39] | payer | Canada  2009 | \| Total cost^a^ (6-month) mean(SD) \| Can$1,379(1,252) \| \| --- \| --- \|   a: family physician visits, medication use, diagnostic tests, health professionals’ visits, specialist visits, herbal remedies, other resources, outpatient visits, emergency department visits, and inpatient visits | €1,762 | |

AN: anorexia nervosa, BED: binge eating disorder, BN: bulimia nervosa, EUR:Euro
